# Newton-type inequalities for the class of modified hyperbolic *p*-convex functions via Riemann-Liouville fractional integrals with numerical justifications

**DOI:** 10.1371/journal.pone.0356882

**Published:** 2026-10-01

**Authors:** Iqra Iqra, Ammara Nosheen, Khuram Ali Khan, Saima Riaz, Michael Kikomba Kahungu

**Affiliations:** 1 Department of Mathematics, University of Sargodha, Sargodha, Pakistan; 2 Higher Institute of Education of Gombe (ISP Gombe), Kinshasa, D. R. Congo; Federal University of Technology - Parana, BRAZIL

## Abstract

In this paper, an identity involving Riemann-Liouville fractional integrals is established which serves as the basis for several new Newton-type inequalities involving the modified class of hyperbolic *p*-convex property of differentiable functions. Some new inequalities are also derived for functions of bounded variation. Examples along with graphs are provided to demonstrate the validity of the newly established inequalities, and the comparison with earlier results highlights their enhanced applicability and significance.

## 1 Introduction

Convex functions are one of the cornerstones of mathematical analysis, with their high level of applicability to a broad class of problems, including optimization theory [1], economic models [2], machine learning algorithms [3], and functional analysis [4]. The basic property that guarantees the uniqueness of the minima for functions is of vital importance for the stability of the methods. The classical concept of convexity has been extremely fruitful, giving rise to a broad class of generalizations, which greatly enrich the mathematical world [5].

Several types of extended convexity notions have been introduced, namely, *s*-convex functions [6], *m*-convex functions [7], hyperbolic *p*-convex functions of a modified class [8], and generalized exponential type convex functions [9]. This idea has been seen to take the most profound form in the theory of inequalities, where results such as Simpson-type inequalities, Newton-type inequalities [10], trapezoidal and midpoint type inequalities, and Giaccardi inequality [11] have been instrumental in solving many scientific and mathematical challenges.

For instance, multi-output quantile regression neural networks [12] use convex optimization algorithms that depend on improved inequalities. Likewise, homogeneity identification in functional coefficient quantile regression with panel data [13] uses convex analysis to accommodate censored data. Quantum physics research involving stabilizer testing and central limit theorems [14] applies convexity in state estimation. Astronomical studies like the detection of new gamma-ray blazars [15] also apply fractional calculus in signal processing.

Fractional calculus has developed into an important mathematical idea with the power to describe the intricate behavior of complex systems, especially those showing memory effects. The increasing use of fractional calculus is an evidence of its power to move beyond the limitations of classical approaches.

The connection between fractional calculus and inequality theory has led to the derivation of several important fractional integral inequalities, which have significant value in the context of approximation theory. These inequalities have important value for the derivation of error estimates for numerical integration schemes, such as trapezoidal, Hermite-Hadamard, Simpson, mid-point, and Ostrowski-type inequalities.

Further developments in this area are the works of Sarikaya and Yildirim [16], who introduced novel Hermite-Hadamard type and midpoint type inequalities for differentiable convex functions in terms of Riemann-Liouville fractional integrals. Sarikaya *et al.* [17] derived generalized Simpson-type inequalities for differentiable *s*-convex functions. Each of the fractional operators has unique properties that make them useful in modeling and analyzing problems in different fields of science and engineering. The fractional order α in the Riemann-Liouville integrals provides additional flexibility, enabling the inequalities to capture memory effects and non-local behavior inherent in many physical and engineering systems.

Recently, several studies have advanced fractional Newton-type inequalities. Meftah et al. [18] established new local fractional Newton-type inequalities. Hezenci and Budak [19] derived fractional Newton-type integral inequalities for various function classes. Özdemir and Yıldız [20] obtained integral inequalities via Caputo and Liouville fractional operators for *m*-convex functions. Karagözoğlu et al. [21] extended Newton-type inequalities to co-ordinated convex functions. Our work complements these by focusing on modified hyperbolic *p*-convex functions.

The research of Simpson-type inequalities within the framework of fractional calculus has gained significant scholarly interest. The authors of [22] derived Simpson-type inequalities for general convex functions in terms of Riemann-Liouville fractional integrals. Later, Chen and Huang [23] provided alternative forms of the inequalities for differentiable *s*-convex functions. Simpson-type and Newton-type inequalities stem from Simpson’s rules [24]:

(i) The following is the Simpson’s quadrature formula, also known as the Simpson’s 1/3 rule.


$$\begin{array}{c} \int_{\kappa_{1}}^{\kappa_{2}}ϝ(\vartheta )d\vartheta \approx \frac{\kappa_{2}-\kappa_{1}}{6}\left[ϝ(\kappa_{1})+4ϝ\left(\frac{\kappa_{1}+\kappa_{2}}{2}\right)+ϝ(\kappa_{2})\right]. \end{array}$$

(ii) The Newton-Cotes quadrature formula or the Simpson’s 3/8 rule.


$$\begin{array}{c} \int_{\kappa_{1}}^{\kappa_{2}}ϝ(\vartheta )d\vartheta \approx \frac{\kappa_{2}-\kappa_{1}}{8}\left[ϝ(\kappa_{1})+3ϝ\left(\frac{2\kappa_{1}+\kappa_{2}}{3}\right)+3ϝ\left(\frac{\kappa_{1}+2\kappa_{2}}{3}\right)+ϝ(\kappa_{2})\right]. \end{array}$$

In [23], the estimations of Simpson-type and Newton-type inequalities are given by the following theorems.

**Theorem 1.1.** *Assume that* $ϝ:[\kappa_{1},\kappa_{2}]\to \mathbb{R}$ *is a mapping that is continuous and four times differentiable on* $\left(\kappa_{1},\kappa_{2}\right)$ *and* $\|{ϝ}^{\left(4\right)}{\|}_{\infty }=\sup_{\vartheta \in (\kappa_{1},\kappa_{2})}|{ϝ}^{\left(4\right)}(\vartheta )|<\infty$*. Then the inequality follows*


$$\begin{array}{c} \left|\frac{1}{6}\left[ϝ(\kappa_{1})+4ϝ\left(\frac{\kappa_{1}+\kappa_{2}}{2}\right)+ϝ(\kappa_{2})\right]-\frac{1}{\kappa_{2}-\kappa_{1}}\int_{\kappa_{1}}^{\kappa_{2}}ϝ(\vartheta )d\vartheta \right|\leq \frac{1}{2880}\|{ϝ}^{\left(4\right)}{\|}_{\infty }(\kappa_{2}-\kappa_{1}{)}^{4}. \end{array}$$

**Theorem 1.2.** *Assume that* $ϝ:[\kappa_{1},\kappa_{2}]\to \mathbb{R}$ *is a four times differentiable, continuous mapping on* $\left(\kappa_{1},\kappa_{2}\right)$ *and* $\|{ϝ}^{\left(4\right)}{\|}_{\infty }=\sup_{\vartheta \in (\kappa_{1},\kappa_{2})}|{ϝ}^{\left(4\right)}(\vartheta )|<\infty$*. Then one has the inequality*


$$\begin{array}{cc} & |\frac{1}{8}\left[ϝ(\kappa_{1})+3ϝ\left(\frac{2\kappa_{1}+\kappa_{2}}{3}\right)+3ϝ\left(\frac{\kappa_{1}+2\kappa_{2}}{3}\right)+ϝ(\kappa_{2})\right]\\ & \;-\frac{1}{\kappa_{2}-\kappa_{1}}\int_{\kappa_{1}}^{\kappa_{2}}ϝ(\vartheta )d\vartheta |\leq \frac{1}{6480}\|{ϝ}^{\left(4\right)}{\|}_{\infty }(\kappa_{2}-\kappa_{1}{)}^{4}. \end{array}$$

The study of Newton-type inequalities for fractional integrals is not merely a mathematical generalization but has practical implications in numerical analysis, error estimation, and signal processing. Modified hyperbolic *p*-convex functions, introduced recently in [8], arise naturally in problems involving hyperbolic geometries and certain nonlinear models where classical convexity fails. However, no fractional Newton-type inequalities have been derived for this class. Our work fills this gap. Furthermore, compared to classical convexity, the additional parameters *m*, *s*, *p* allow finer control over function growth, making the inequalities adaptable to a wider range of applications, such as fractional-order system identification and machine learning regularization. The numerical comparisons with [25] demonstrate that our bounds are often tighter, providing better error estimates for quadrature rules.

Motivated by our ongoing research, we derive Newton-type inequalities for differentiable modified hyperbolic *p*-convex functions and functions of bounded variation in terms of Riemann-Liouville fractional integrals. Moreover, we illustrate their validity with graphical examples.

The paper is organized as follows: section 2 includes some fundamental definitions of fractional calculus and some preliminary important results. One important integral identity which plays a central role to prove our main results is established in section 3 and we present new Newton-type inequalities for Riemann-Liouville fractional integrals of modified hyperbolic *p*-convex functions. Using Riemann-Liouville fractional integrals, we establish fractional Newton-type inequalities for functions of bounded variation in section 4. The paper is concluded in section 5 by giving some ideas for further studies.

## 2 Preliminaries

**Definition 2.2. (Total variation).** *Let* $ϝ:[\kappa_{1},\kappa_{2}]\to \mathbb{R}$ *be a function. The total variation of* ϝ *over* $\left[\kappa_{1},\kappa_{2}\right]$ *is defined as*


$$\begin{array}{c} \overset{\kappa_{2}}{\underset{\kappa_{1}}{⋁}}(ϝ)=\sup\sum_{i=1}^{n}|ϝ(x_{i})-ϝ(x_{i-1})|, \end{array}$$

*where the supremum is taken over all partitions* $\kappa_{1}=x_{0}<x_{1}<\cdot \cdot \cdot <x_{n}=\kappa_{2}$*. If* $\overset{\kappa_{2}}{\underset{\kappa_{1}}{⋁}}(ϝ)<\infty$*, then* ϝ *is said to be of bounded variation on* $\left[\kappa_{1},\kappa_{2}\right]$.

**Definition 2.3. (Modified hyperbolic** *p***-convex function).** [8] *A function* ϝ:ℑ⊆ℝ→ℝ *is called modified hyperbolic p-convex on* ℑ *if for any closed subinterval* $\left[\kappa_{1},\kappa_{2}\right]$ *of* ℑ*, we have*


$$\begin{array}{ccc} ϝ\left({\left[\vartheta \kappa_{1}^{p}+m(1-\vartheta )\kappa_{2}^{p}\right]}^{\frac{1}{p}}\right) & \leq & \frac{\sinh\left[p(1-s(1-\vartheta ))(\kappa_{2}-\kappa_{1})\right]}{\sinh\left[p(\kappa_{2}-\kappa_{1})\right]}ϝ(\kappa_{1})\\ & + & m\frac{\sinh\left[p(1-s\vartheta )(\kappa_{2}-\kappa_{1})\right]}{\sinh\left[p(\kappa_{2}-\kappa_{1})\right]}ϝ(\kappa_{2}), \end{array}$$ (1)

*∀* m∈[0,1],s∈[0,1],ϑ∈[0,1] *and* $p\in {\mathbb{R}}^{+}$.

**Definition 2.4. (Riemann-Liouville fractional integrals).** [26] *Let* $ϝ:[\kappa_{1},\kappa_{2}]\to \mathbb{R}$ *be an integrable function. Then the Riemann-Liouville fractional integrals* ${ℑ}_{\kappa_{1}+}^{\alpha }ϝ(x)$ *and* ${ℑ}_{\kappa_{2}-}^{\alpha }ϝ(x)$ *of order* α>0 *are defined as follows*


$$\begin{array}{c} {ℑ}_{\kappa_{1}+}^{\alpha }ϝ(x)=\frac{1}{\Gamma (\alpha )}\int_{\kappa_{1}}^{x}(x-\vartheta {)}^{\alpha -1}ϝ(\vartheta )d\vartheta ,\;x>\kappa_{1}, \end{array}$$

*and*


$$\begin{array}{c} {ℑ}_{\kappa_{2}-}^{\alpha }ϝ(x)=\frac{1}{\Gamma (\alpha )}\int_{x}^{\kappa_{2}}(\vartheta -x{)}^{\alpha -1}ϝ(\vartheta )d\vartheta ,\;x<\kappa_{2}, \end{array}$$

*where* Γ(·) *represents the well-known Euler gamma function.*

**Theorem 2.4.** *[27] (Hölder’s integral inequality) If* ϝ *and G are real functions defined on* $\left[\kappa_{1},\kappa_{2}\right]$ *and* $|ϝ{|}^{v},|G{|}^{t}$ *are integrable functions on* $\left[\kappa_{1},\kappa_{2}\right]$*, then*


$$\begin{array}{c} \int_{\kappa_{1}}^{\kappa_{2}}|ϝ(\vartheta )G(\vartheta )|d\vartheta \leq {\left(\int_{\kappa_{1}}^{\kappa_{2}}|ϝ(\vartheta ){|}^{v}d\vartheta \right)}^{\frac{1}{v}}{\left(\int_{\kappa_{1}}^{\kappa_{2}}|G(\vartheta ){|}^{t}d\vartheta \right)}^{\frac{1}{t}}, \end{array}$$

*where v > 1 and* $\frac{1}{v}+\frac{1}{t}=1$.

**Theorem 2.5.** *[28] (Power-mean integral inequality) Let* v≥1*. If* ϝ *and G are real functions defined on* $\left[\kappa_{1},\kappa_{2}\right]$ *and if* $|ϝ|,|ϝ||G{|}^{v}$ *are integrable functions on* $\left[\kappa_{1},\kappa_{2}\right]$*, then*


$$\begin{array}{c} \int_{\kappa_{1}}^{\kappa_{2}}|ϝ(\vartheta )G(\vartheta )|d\vartheta \leq {\left(\int_{\kappa_{1}}^{\kappa_{2}}|ϝ(\vartheta )|d\vartheta \right)}^{1-\frac{1}{v}}{\left(\int_{\kappa_{1}}^{\kappa_{2}}|ϝ(\vartheta )||G(\vartheta ){|}^{v}d\vartheta \right)}^{\frac{1}{v}}. \end{array}$$

Alomari [29] established the inequality mentioned below for functions of bounded variation.

**Theorem 2.6.** *Let* $ϝ:[\kappa_{1},\kappa_{2}]\to \mathbb{R}$ *be a mapping of bounded variation on* $\left[\kappa_{1},\kappa_{2}\right]$*. Then, for all* λ∈[0,1] *and*


$$\begin{array}{c} \kappa_{1}+\lambda \frac{\kappa_{2}-\kappa_{1}}{2}\leq x\leq \frac{\kappa_{1}+\kappa_{2}}{2}, \end{array}$$

*the following inequality is true.*


$$\begin{array}{cc} & \left|(\kappa_{2}-\kappa_{1})\left[\lambda \frac{ϝ(\kappa_{1})+ϝ(\kappa_{2})}{2}+(1-\lambda )\frac{ϝ(x)+ϝ(\kappa_{1}+\kappa_{2}-x)}{2}\right]-\int_{\kappa_{1}}^{\kappa_{2}}ϝ(\vartheta )d\vartheta \right|\\ & \leq \max\left\{\lambda \frac{\kappa_{2}-\kappa_{1}}{2},\left(x-\frac{(2-\lambda )\kappa_{1}+\lambda \kappa_{2}}{2}\right),\left(\frac{\kappa_{1}+\kappa_{2}}{2}-x\right)\right\}\overset{\kappa_{2}}{\underset{\kappa_{1}}{⋁}}(ϝ). \end{array}$$

Soontharanon *et al.* [30] proved the given fractional Newton-type inequality for functions of bounded variation.

**Theorem 2.7.** *Let* $ϝ:[\kappa_{1},\kappa_{2}]\to \mathbb{R}$ *be a function of bounded variation on* $\left[\kappa_{1},\kappa_{2}\right]$*. Then the following fractional Newton-type inequality for generalized fractional integrals is valid.*


$$\begin{array}{cc} & |\frac{1}{3𝒯(1)}\left[{ℑ}_{\kappa_{1}+}^{\alpha }\;ϝ\left(\frac{2\kappa_{1}+\kappa_{2}}{3}\right)+{ℑ}_{\frac{2\kappa_{1}+\kappa_{2}}{3}+}^{\alpha }\;ϝ\left(\frac{\kappa_{1}+2\kappa_{2}}{3}\right)+{ℑ}_{\frac{\kappa_{1}+2\kappa_{2}}{3}+}^{\alpha }\;ϝ\left(\kappa_{2}\right)\right]\\ & -\frac{1}{8}\left[ϝ(\kappa_{1})+3ϝ\left(\frac{2\kappa_{1}+\kappa_{2}}{3}\right)+3ϝ\left(\frac{\kappa_{1}+2\kappa_{2}}{3}\right)+ϝ(\kappa_{2})\right]|\\ & \leq \frac{5}{24}\overset{\kappa_{2}}{\underset{\kappa_{1}}{⋁}}(ϝ), \end{array}$$

*where*


$$\begin{array}{c} 𝒯(x)=\int_{0}^{x}\frac{\phi (((\kappa_{2}-\kappa_{1})/3)\vartheta )}{\vartheta }d\vartheta <+\infty , \end{array}$$

*and* ϕ *is a function such that the integral converges.*

## 3 Main results

In this section, we first prove an integral identity that will serve as the foundation for our main results. Then, using the definition of modified hyperbolic *p*-convex functions along with classical inequalities such as Hölder and power-mean, we establish several new Newton-type inequalities. Numerical examples and graphical illustrations are provided to demonstrate the validity of the obtained results, and a comparison with existing work shows that our bounds are often tighter.

For the sake of brevity, the following notations will be adopted.


$$\begin{array}{c} \begin{array}{c} A_{1}=\frac{1}{m(2\kappa_{1}+\kappa_{2}{)}^{p}-(3\kappa_{1}{)}^{p}},\\ A_{2}=\frac{1}{m(\kappa_{1}+2\kappa_{2}{)}^{p}-(2\kappa_{1}+\kappa_{2}{)}^{p}},\\ A_{3}=\frac{1}{m(3\kappa_{2}{)}^{p}-(\kappa_{1}+2\kappa_{2}{)}^{p}}, \end{array}\} \end{array}$$ (2)

where $\kappa_{1},\kappa_{2}\in ℑ\subseteq [0,\infty )$, and *p* > 0.

**Lemma 3.1.** *Let the mapping* ϝ:ℑ⊆[0,∞)→ℝ *be differentiable over the interior* ${ℑ}^{o}$ *with* $\kappa_{1},\kappa_{2}\in ℑ$*,* $\kappa_{1}<\kappa_{2}$ *and* $ϝ\in L_{1}[\kappa_{1},\kappa_{2}]$*. Then the following identity holds.*


$$\begin{array}{cc} & (3{)}^{p(\alpha -1)}\;\Gamma (\alpha +1)[A_{1}^{\alpha +1}\;{ℑ}_{\kappa_{1}+}^{\alpha }\;ϝ\left(m\left(\frac{2\kappa_{1}+\kappa_{2}}{3}\right)\right)+A_{2}^{\alpha +1}\;{ℑ}_{\frac{2\kappa_{1}+\kappa_{2}}{3}+}^{\alpha }\;ϝ\left(m\left(\frac{\kappa_{1}+2\kappa_{2}}{3}\right)\right)\\ & +A_{3}^{\alpha +1}\;{ℑ}_{\frac{\kappa_{1}+2\kappa_{2}}{3}+}^{\alpha }\;ϝ\left(m\kappa_{2}\right)]-\frac{1}{8}[3A_{1}ϝ(\kappa_{1})+5A_{1}ϝ\left(m\left(\frac{2\kappa_{1}+\kappa_{2}}{3}\right)\right)+4A_{2}ϝ\left(\frac{2\kappa_{1}+\kappa_{2}}{3}\right)\\ & +4A_{2}ϝ\left(m\left(\frac{\kappa_{1}+2\kappa_{2}}{3}\right)\right)+5A_{3}ϝ\left(\frac{\kappa_{1}+2\kappa_{2}}{3}\right)+3A_{3}ϝ(m\kappa_{2})]\\ & =\frac{1}{3^{p}(p)}\left[I_{1}+I_{2}+I_{3}\right], \end{array}$$ (3)

*where A*_*1*_*, A*_*2*_*, and A*_*3*_ *are defined in (2) and*


$$\begin{array}{cc} & I_{1}=\int_{0}^{1}\left(\vartheta^{\alpha }-\frac{5}{8}\right)\frac{{ϝ}^{{\;}^{\prime }}\left({\left[\vartheta \kappa_{1}^{p}+m(1-\vartheta ){\left(\frac{2\kappa_{1}+\kappa_{2}}{3}\right)}^{p}\right]}^{\frac{1}{p}}\right)}{{\left[\vartheta \kappa_{1}^{p}+m(1-\vartheta )(\frac{2\kappa_{1}+\kappa_{2}}{3}{)}^{p}\right]}^{1-\frac{1}{p}}}d\vartheta ,\\ & I_{2}=\int_{0}^{1}\left(\vartheta^{\alpha }-\frac{1}{2}\right)\frac{{ϝ}^{{\;}^{\prime }}\left({\left[\vartheta {\left(\frac{2\kappa_{1}+\kappa_{2}}{3}\right)}^{p}+m(1-\vartheta ){\left(\frac{\kappa_{1}+2\kappa_{2}}{3}\right)}^{p}\right]}^{\frac{1}{p}}\right)}{{\left[\vartheta (\frac{2\kappa_{1}+\kappa_{2}}{3}{)}^{p}+m(1-\vartheta )(\frac{\kappa_{1}+2\kappa_{2}}{3}{)}^{p}\right]}^{1-\frac{1}{p}}}d\vartheta ,\\ & I_{3}=\int_{0}^{1}\left(\vartheta^{\alpha }-\frac{3}{8}\right)\frac{{ϝ}^{{\;}^{\prime }}\left({\left[\vartheta {\left(\frac{\kappa_{1}+2\kappa_{2}}{3}\right)}^{p}+m(1-\vartheta )\kappa_{2}^{p}\right]}^{\frac{1}{p}}\right)}{{\left[\vartheta (\frac{\kappa_{1}+2\kappa_{2}}{3}{)}^{p}+m(1-\vartheta )\kappa_{2}^{p}\right]}^{1-\frac{1}{p}}}d\vartheta . \end{array}$$

*Proof.* Integrating by parts and using change of variables, we obtain


$$\begin{array}{cc} I_{1}= & \int_{0}^{1}\left(\vartheta^{\alpha }-\frac{5}{8}\right)\frac{{ϝ}^{{\;}^{\prime }}\left({\left[\vartheta \kappa_{1}^{p}+m(1-\vartheta ){\left(\frac{2\kappa_{1}+\kappa_{2}}{3}\right)}^{p}\right]}^{\frac{1}{p}}\right)}{{\left[\vartheta \kappa_{1}^{p}+m(1-\vartheta )(\frac{2\kappa_{1}+\kappa_{2}}{3}{)}^{p}\right]}^{1-\frac{1}{p}}}d\vartheta \\ = & {\left(3^{p}\right)}^{\alpha +1}p^{2}\Gamma (\alpha +1)A_{1}^{\alpha +1}\;{ℑ}_{\kappa_{1}+}^{\alpha }\;ϝ\left(m\left(\frac{2\kappa_{1}+\kappa_{2}}{3}\right)\right)\\ & -\frac{3^{p}(p)}{8}\left[3A_{1}ϝ(\kappa_{1})+5A_{1}ϝ\left(m\left(\frac{2\kappa_{1}+\kappa_{2}}{3}\right)\right)\right]. \end{array}$$ (4)

$$\begin{array}{cc} I_{2}= & \int_{0}^{1}\left(\vartheta^{\alpha }-\frac{1}{2}\right)\frac{{ϝ}^{{\;}^{\prime }}\left({\left[\vartheta {\left(\frac{2\kappa_{1}+\kappa_{2}}{3}\right)}^{p}+m(1-\vartheta ){\left(\frac{\kappa_{1}+2\kappa_{2}}{3}\right)}^{p}\right]}^{\frac{1}{p}}\right)}{{\left[\vartheta (\frac{2\kappa_{1}+\kappa_{2}}{3}{)}^{p}+m(1-\vartheta )(\frac{\kappa_{1}+2\kappa_{2}}{3}{)}^{p}\right]}^{1-\frac{1}{p}}}d\vartheta \\ = & {\left(3^{p}\right)}^{\alpha +1}p^{2}\Gamma (\alpha +1)A_{2}^{\alpha +1}\;{ℑ}_{\frac{2\kappa_{1}+\kappa_{2}}{3}+}^{\alpha }\;ϝ\left(m\left(\frac{\kappa_{1}+2\kappa_{2}}{3}\right)\right)\\ & -\frac{3^{p}(p)}{8}\left[4A_{2}ϝ\left(m\left(\frac{2\kappa_{1}+\kappa_{2}}{3}\right)\right)+4A_{2}ϝ\left(m\left(\frac{\kappa_{1}+2\kappa_{2}}{3}\right)\right)\right], \end{array}$$ (5)

and


$$\begin{array}{cc} I_{3}= & \int_{0}^{1}\left(\vartheta^{\alpha }-\frac{3}{8}\right)\frac{{ϝ}^{{\;}^{\prime }}\left({\left[\vartheta {\left(\frac{\kappa_{1}+2\kappa_{2}}{3}\right)}^{p}+m(1-\vartheta )\kappa_{2}^{p}\right]}^{\frac{1}{p}}\right)}{{\left[\vartheta (\frac{\kappa_{1}+2\kappa_{2}}{3}{)}^{p}+m(1-\vartheta )\kappa_{2}^{p}\right]}^{1-\frac{1}{p}}}d\vartheta \\ = & {\left(3^{p}\right)}^{\alpha +1}p^{2}\Gamma (\alpha +1)A_{3}^{\alpha +1}\;{ℑ}_{\frac{\kappa_{1}+2\kappa_{2}}{3}+}^{\alpha }\;ϝ(m\kappa_{2})\\ & -\frac{3^{p}(p)}{8}\left[5A_{3}ϝ\left(m\left(\frac{\kappa_{1}+2\kappa_{2}}{3}\right)\right)+3A_{3}ϝ(m\kappa_{2})\right]. \end{array}$$ (6)

Hence, adding (4)-(6) and multiplying by $\frac{1}{3^{p}(p)}$ yields the required identity. □

**Remark 3.2.** *By substituting p = 1 and m = 1 in* (3)*, it reduces to Lemma 3.1 of [25].*

**Theorem 3.3.** *Assume that the conditions of Lemma 3.1 hold. If* $\left|{ϝ}^{{\;}^{\prime }}\right|$ *is modified hyperbolic p-convex on* $\left[\kappa_{1},\kappa_{2}\right]$*, then the following Newton-type inequality holds.*


$$\begin{array}{cc} & |(3{)}^{p(\alpha -1)}\;\Gamma (\alpha +1)[A_{1}^{\alpha +1}\;{ℑ}_{\kappa_{1}+}^{\alpha }\;ϝ\left(m\left(\frac{2\kappa_{1}+\kappa_{2}}{3}\right)\right)+A_{2}^{\alpha +1}\;{ℑ}_{\frac{2\kappa_{1}+\kappa_{2}}{3}+}^{\alpha }\;ϝ\left(m\left(\frac{\kappa_{1}+2\kappa_{2}}{3}\right)\right)\\ & +A_{3}^{\alpha +1}\;{ℑ}_{\frac{\kappa_{1}+2\kappa_{2}}{3}+}^{\alpha }\;ϝ\left(m\kappa_{2}\right)]-\frac{1}{8}[3A_{1}ϝ(\kappa_{1})+5A_{1}ϝ\left(m\left(\frac{2\kappa_{1}+\kappa_{2}}{3}\right)\right)+4A_{2}ϝ\left(m\left(\frac{2\kappa_{1}+\kappa_{2}}{3}\right)\right)\\ & +4A_{2}ϝ\left(m\left(\frac{\kappa_{1}+2\kappa_{2}}{3}\right)\right)+5A_{3}ϝ\left(\frac{\kappa_{1}+2\kappa_{2}}{3}\right)+3A_{3}ϝ(m\kappa_{2})]|\\ & \leq \frac{1}{3^{p}(p)}[B_{1}\left|{ϝ}^{{\;}^{\prime }}(\kappa_{1})\right|+mB_{2}\left|{ϝ}^{{\;}^{\prime }}\left(\frac{2\kappa_{1}+\kappa_{2}}{3}\right)\right|+B_{3}\left|{ϝ}^{{\;}^{\prime }}\left(\frac{2\kappa_{1}+\kappa_{2}}{3}\right)\right|+mB_{4}\left|{ϝ}^{{\;}^{\prime }}\left(\frac{\kappa_{1}+2\kappa_{2}}{3}\right)\right|\\ & +B_{5}\left|{ϝ}^{{\;}^{\prime }}\left(\frac{\kappa_{1}+2\kappa_{2}}{3}\right)\right|+B_{6}\left|{ϝ}^{{\;}^{\prime }}(\kappa_{2})\right|], \end{array}$$ (7)

*where A*_*1*_*, A*_*2*_*, and A*_*3*_ *are given in (2) and*


$$\begin{array}{cc} & B_{1}=\int_{0}^{1}\frac{\sinh\left[p\left(1-s(1-\vartheta )\right)\left(\frac{k_{2}-k_{1}}{3}\right)\right]\left|\vartheta^{\alpha }-\frac{5}{8}\right|}{\sinh\left[p\left(\frac{k_{2}-k_{1}}{3}\right)\right]{\left[\vartheta (\kappa_{1}{)}^{p}+m(1-\vartheta )(\frac{2\kappa_{1}+\kappa_{2}}{3}{)}^{p}\right]}^{1-\frac{1}{p}}}d\vartheta ,\\ & B_{2}=\int_{0}^{1}\frac{\sinh\left[p\left(1-s\vartheta \right)\left(\frac{k_{2}-k_{1}}{3}\right)\right]\left|\vartheta^{\alpha }-\frac{5}{8}\right|}{\sinh\left[p\left(\frac{k_{2}-k_{1}}{3}\right)\right]{\left[\vartheta (\kappa_{1}{)}^{p}+m(1-\vartheta )(\frac{2\kappa_{1}+\kappa_{2}}{3}{)}^{p}\right]}^{1-\frac{1}{p}}}d\vartheta ,\\ & B_{3}=\int_{0}^{1}\frac{\sinh\left[p\left(1-s(1-\vartheta )\right)\left(\frac{k_{2}-k_{1}}{3}\right)\right]\left|\vartheta^{\alpha }-\frac{1}{2}\right|}{\sinh\left[p\left(\frac{k_{2}-k_{1}}{3}\right)\right]{\left[\vartheta (\frac{2\kappa_{1}+\kappa_{2}}{3}{)}^{p}+m(1-\vartheta )(\frac{\kappa_{1}+2\kappa_{2}}{3}{)}^{p}\right]}^{1-\frac{1}{p}}}d\vartheta ,\\ & B_{4}=\int_{0}^{1}\frac{\sinh\left[p\left(1-s\vartheta \right)\left(\frac{k_{2}-k_{1}}{3}\right)\right]\left|\vartheta^{\alpha }-\frac{1}{2}\right|}{\sinh\left[p\left(\frac{k_{2}-k_{1}}{3}\right)\right]{\left[\vartheta (\frac{2\kappa_{1}+\kappa_{2}}{3}{)}^{p}+m(1-\vartheta )(\frac{\kappa_{1}+2\kappa_{2}}{3}{)}^{p}\right]}^{1-\frac{1}{p}}}d\vartheta ,\\ & B_{5}=\int_{0}^{1}\frac{\sinh\left[p\left(1-s(1-\vartheta )\right)\left(\frac{k_{2}-k_{1}}{3}\right)\right]\left|\vartheta^{\alpha }-\frac{3}{8}\right|}{\sinh\left[p\left(\frac{k_{2}-k_{1}}{3}\right)\right]{\left[\vartheta (\frac{\kappa_{1}+2\kappa_{2}}{3}{)}^{p}+m(1-\vartheta )(\kappa_{2}{)}^{p}\right]}^{1-\frac{1}{p}}}d\vartheta ,\\ & B_{6}=\int_{0}^{1}\frac{\sinh\left[p\left(1-s\vartheta \right)\left(\frac{k_{2}-k_{1}}{3}\right)\right]\left|\vartheta^{\alpha }-\frac{3}{8}\right|}{\sinh\left[p\left(\frac{k_{2}-k_{1}}{3}\right)\right]{\left[\vartheta (\frac{\kappa_{1}+2\kappa_{2}}{3}{)}^{p}+m(1-\vartheta )(\kappa_{2}{)}^{p}\right]}^{1-\frac{1}{p}}}d\vartheta . \end{array}$$

*Proof.* By using the property of absolute value on (3) and modified hyperbolic *p*-convexity of $\left|{ϝ}^{{\;}^{\prime }}\right|$, it follows that


$$\begin{array}{cc} & |(3{)}^{p(\alpha -1)}\;\Gamma (\alpha +1)[A_{1}^{\alpha +1}\;{ℑ}_{\kappa_{1}+}^{\alpha }\;ϝ\left(m\left(\frac{2\kappa_{1}+\kappa_{2}}{3}\right)\right)+A_{2}^{\alpha +1}\;{ℑ}_{\frac{2\kappa_{1}+\kappa_{2}}{3}+}^{\alpha }\;ϝ\left(m\left(\frac{\kappa_{1}+2\kappa_{2}}{3}\right)\right)\\ & +A_{3}^{\alpha +1}\;{ℑ}_{\frac{\kappa_{1}+2\kappa_{2}}{3}+}^{\alpha }\;ϝ\left(m\kappa_{2}\right)]-\frac{1}{8}[3A_{1}ϝ(\kappa_{1})+5A_{1}ϝ\left(m\left(\frac{2\kappa_{1}+\kappa_{2}}{3}\right)\right)+4A_{2}ϝ\left(m\left(\frac{2\kappa_{1}+\kappa_{2}}{3}\right)\right)\\ & +4A_{2}ϝ\left(m\left(\frac{\kappa_{1}+2\kappa_{2}}{3}\right)\right)+5A_{3}ϝ\left(\frac{\kappa_{1}+2\kappa_{2}}{3}\right)+3A_{3}ϝ(m\kappa_{2})]|\\ & \leq \frac{1}{3^{p}(p)}[\int_{0}^{1}\left|\vartheta^{\alpha }-\frac{5}{8}\right|\frac{\left|{ϝ}^{{\;}^{\prime }}\left({\left[\vartheta (\kappa_{1}{)}^{p}+m(1-\vartheta ){\left(\frac{2\kappa_{1}+\kappa_{2}}{3}\right)}^{p}\right]}^{\frac{1}{p}}\right)\right|}{{\left[\vartheta (\kappa_{1}{)}^{p}+m(1-\vartheta )(\frac{2\kappa_{1}+\kappa_{2}}{3}{)}^{p}\right]}^{1-\frac{1}{p}}}d\vartheta \\ & +\int_{0}^{1}\left|\vartheta^{\alpha }-\frac{1}{2}\right|\frac{\left|{ϝ}^{{\;}^{\prime }}\left({\left[\vartheta {\left(\frac{2\kappa_{1}+\kappa_{2}}{3}\right)}^{p}+m(1-\vartheta ){\left(\frac{\kappa_{1}+2\kappa_{2}}{3}\right)}^{p}\right]}^{\frac{1}{p}}\right)\right|}{{\left[\vartheta (\frac{2\kappa_{1}+\kappa_{2}}{3}{)}^{p}+m(1-\vartheta )(\frac{\kappa_{1}+2\kappa_{2}}{3}{)}^{p}\right]}^{1-\frac{1}{p}}}d\vartheta \\ & +\int_{0}^{1}\left|\vartheta^{\alpha }-\frac{3}{8}\right|\frac{\left|{ϝ}^{{\;}^{\prime }}\left({\left[\vartheta {\left(\frac{\kappa_{1}+2\kappa_{2}}{3}\right)}^{p}+(1-\vartheta )\kappa_{2}^{p}\right]}^{\frac{1}{p}}\right)\right|}{{\left[\vartheta (\frac{\kappa_{1}+2\kappa_{2}}{3}{)}^{p}+m(1-\vartheta )\kappa_{2}^{p}\right]}^{1-\frac{1}{p}}}d\vartheta ] \end{array}$$

$$\begin{array}{cc} & \leq \frac{1}{3^{p}(p)}[|{ϝ}^{{\;}^{\prime }}(\kappa_{1})|\int_{0}^{1}\frac{\sinh\left[p\left(1-s(1-\vartheta )\right)\left(\frac{k_{2}-k_{1}}{3}\right)\right]\left|\vartheta^{\alpha }-\frac{5}{8}\right|}{\sinh\left[p\left(\frac{k_{2}-k_{1}}{3}\right)\right]{\left[\vartheta (\kappa_{1}{)}^{p}+m(1-\vartheta )(\frac{2\kappa_{1}+\kappa_{2}}{3}{)}^{p}\right]}^{1-\frac{1}{p}}}d\vartheta \\ & +m\left|{ϝ}^{{\;}^{\prime }}\left(\frac{2\kappa_{1}+\kappa_{2}}{3}\right)\right|\int_{0}^{1}\frac{\sinh\left[p\left(1-s\vartheta \right)\left(\frac{k_{2}-k_{1}}{3}\right)\right]\left|\vartheta^{\alpha }-\frac{5}{8}\right|}{\sinh\left[p\left(\frac{k_{2}-k_{1}}{3}\right)\right]{\left[\vartheta (\kappa_{1}{)}^{p}+m(1-\vartheta ){\left(\frac{2\kappa_{1}+\kappa_{2}}{3}\right)}^{p}\right]}^{1-\frac{1}{p}}}d\vartheta \\ & +\left|{ϝ}^{{\;}^{\prime }}\left(\frac{2\kappa_{1}+\kappa_{2}}{3}\right)\right|\int_{0}^{1}\frac{\sinh\left[p\left(1-s(1-\vartheta )\right)\left(\frac{k_{2}-k_{1}}{3}\right)\right]\left|\vartheta^{\alpha }-\frac{1}{2}\right|}{\sinh\left[p\left(\frac{k_{2}-k_{1}}{3}\right)\right]{\left[\vartheta (\frac{2\kappa_{1}+\kappa_{2}}{3}{)}^{p}+m(1-\vartheta ){\left(\frac{\kappa_{1}+2\kappa_{2}}{3}\right)}^{p}\right]}^{1-\frac{1}{p}}}d\vartheta \\ & +m\left|{ϝ}^{{\;}^{\prime }}\left(\frac{\kappa_{1}+2\kappa_{2}}{3}\right)\right|\int_{0}^{1}\frac{\sinh\left[p\left(1-s\vartheta \right)\left(\frac{k_{2}-k_{1}}{3}\right)\right]\left|\vartheta^{\alpha }-\frac{1}{2}\right|}{\sinh\left[p\left(\frac{k_{2}-k_{1}}{3}\right)\right]{\left[\vartheta (\frac{2\kappa_{1}+\kappa_{2}}{3}{)}^{p}+m(1-\vartheta )(\frac{\kappa_{1}+2\kappa_{2}}{3}{)}^{p}\right]}^{1-\frac{1}{p}}}d\vartheta \\ & +\left|{ϝ}^{{\;}^{\prime }}\left(\frac{\kappa_{1}+2\kappa_{2}}{3}\right)\right|\int_{0}^{1}\frac{\sinh\left[p\left(1-s(1-\vartheta )\right)\left(\frac{k_{2}-k_{1}}{3}\right)\right]\left|\vartheta^{\alpha }-\frac{3}{8}\right|}{\sinh\left[p\left(\frac{k_{2}-k_{1}}{3}\right)\right]{\left[\vartheta (\frac{\kappa_{1}+2\kappa_{2}}{3}{)}^{p}+m(1-\vartheta )\kappa_{2}^{p}\right]}^{1-\frac{1}{p}}}d\vartheta \\ & +m|{ϝ}^{{\;}^{\prime }}(\kappa_{2})|\int_{0}^{1}\frac{\sinh\left[p\left(1-s\vartheta \right)\left(\frac{k_{2}-k_{1}}{3}\right)\right]\left|\vartheta^{\alpha }-\frac{3}{8}\right|}{\sinh\left[p\left(\frac{k_{2}-k_{1}}{3}\right)\right]{\left[\vartheta (\frac{\kappa_{1}+2\kappa_{2}}{3}{)}^{p}+m(1-\vartheta )\kappa_{2}^{p}\right]}^{1-\frac{1}{p}}}d\vartheta ]. \end{array}$$

This concludes the proof. □

**Remark 3.4.** *By substituting p = 1, s = 1, m = 1, and* sinh(p𝔱)=p𝔱 *in (7), it reduces to Theorem 4 of [25].*

**Corollary 3.5.** *By substituting* α=1*, p = 1, m = 1, s = 1, and* sinh(p𝔱)=p𝔱 *in Theorem 3.3, the following inequality is obtained:*


$$\begin{array}{cc} & \left|\frac{1}{\kappa_{2}-\kappa_{1}}\int_{\kappa_{1}}^{\kappa_{2}}ϝ(\vartheta )d\vartheta -\frac{1}{8}\left[ϝ(\kappa_{1})+3ϝ\left(\frac{2\kappa_{1}+\kappa_{2}}{3}\right)+3ϝ\left(\frac{\kappa_{1}+2\kappa_{2}}{3}\right)+ϝ(\kappa_{2})\right]\right|\\ & \leq \frac{\kappa_{2}-\kappa_{1}}{13824}\left[157|{ϝ}^{{\;}^{\prime }}(\kappa_{1})|+443\left|{ϝ}^{{\;}^{\prime }}\left(\frac{2\kappa_{1}+\kappa_{2}}{3}\right)\right|+443\left|{ϝ}^{{\;}^{\prime }}\left(\frac{\kappa_{1}+2\kappa_{2}}{3}\right)\right|+251|{ϝ}^{{\;}^{\prime }}(\kappa_{2})|\right]. \end{array}$$

*Equality holds when* ϝ *is a linear function.*

**Example 3.6** *Define a function* ϝ:[0,1]→ℝ *by* $ϝ(\vartheta )=\frac{\vartheta^{3}}{3}$  *such that* ${ϝ}^{{\;}^{\prime }}(\vartheta )=\vartheta^{2}$*, and* $\left|{ϝ}^{{\;}^{\prime }}\right|$ *is modified hyperbolic p-convex on [0,1]. By taking* $\kappa_{2}=1,\kappa_{1}=0,m=1,s=0.5,p=2$*, and* α=0.2*, under these assumptions, inequality (7) reduces to* 0.00726≤0.3241*. Also it is clear from* Figs 1 *and* 2 *that* blueLHS≤redRHS *for all* α>0.

**Fig 1:**
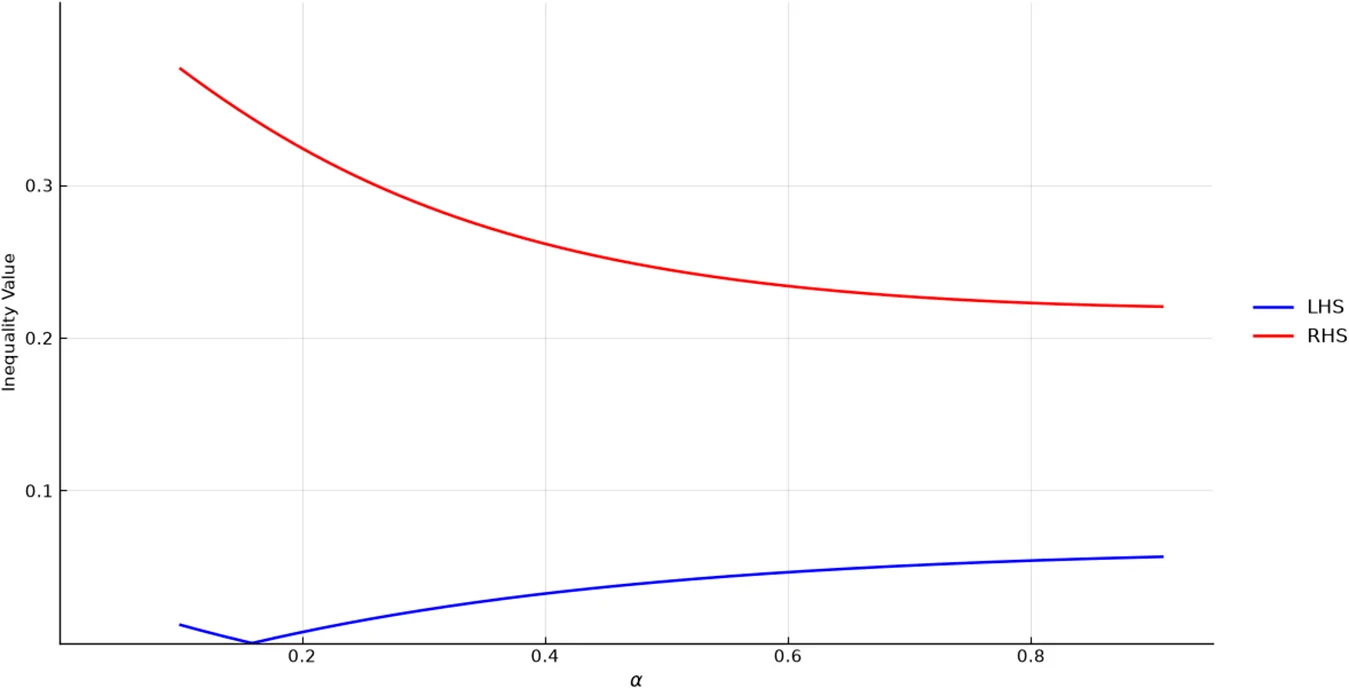
2D graphical representation of Example 3.6 for inequality (7). Horizontal axis: fractional order α, vertical axis: Inequality value. Blue curve: LHS, red curve: RHS. The inequality LHS≤RHS holds for all α>0.

**Fig 2:**
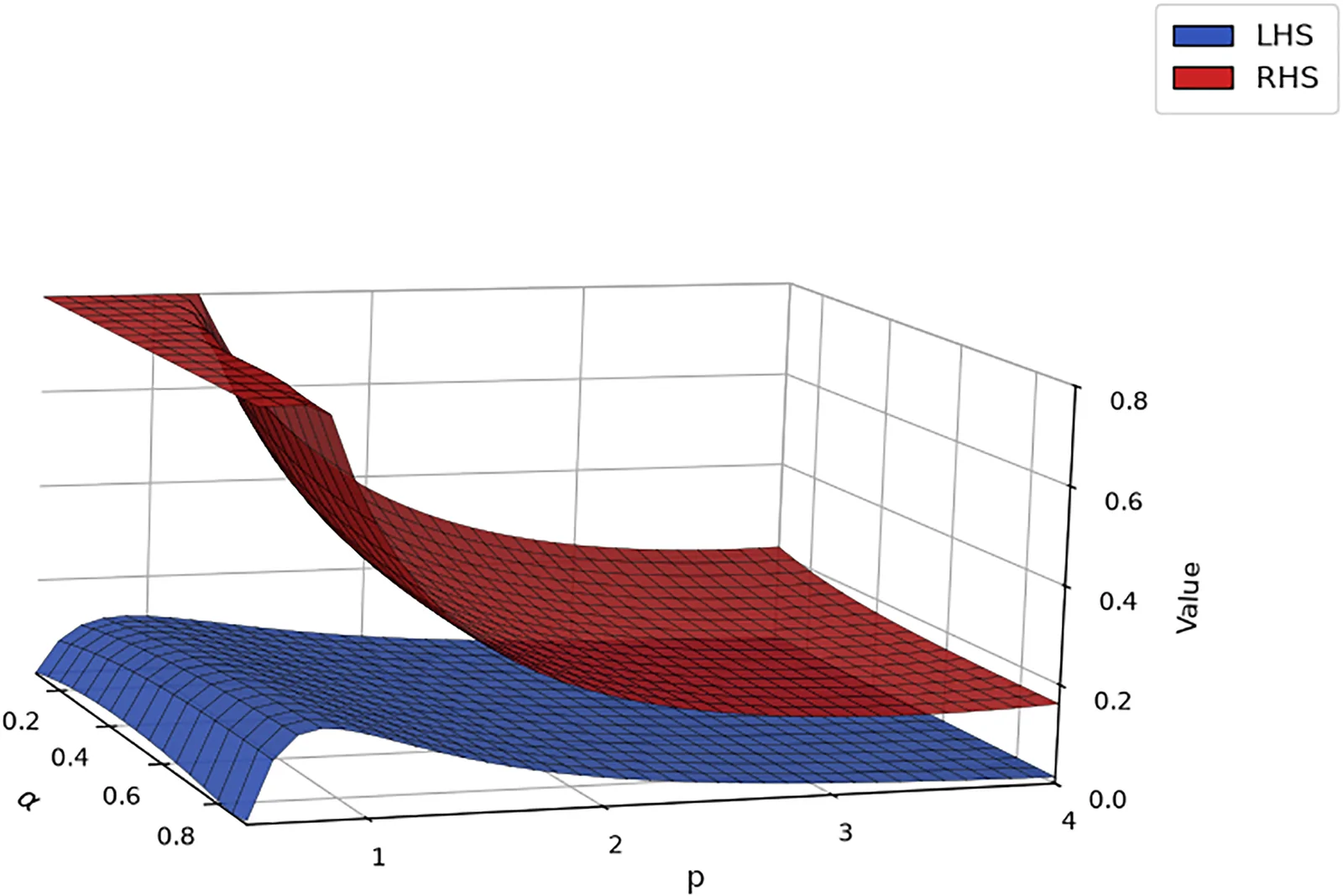
3D graphical representation of Example 3.6. Blue surface: LHS, red surface: RHS as functions of α (horizontal axis) and *p* (depth axis). The red surface lies above the blue surface, confirming the inequality.

Now, Hölder and power-mean inequalities are utilized to obtain some more refined results.

**Theorem 3.7.** *Assume that the requirements of Lemma 3.1 are met. If* $|{ϝ}^{{\;}^{\prime }}{|}^{q}$*, q > 1 and* $\frac{1}{t}+\frac{1}{q}=1$*, is modified hyperbolic p-convex function, then the following Newton-type inequality holds true.*


$$\begin{array}{cc} & |(3{)}^{p(\alpha -1)}\;\Gamma (\alpha +1)[A_{1}^{\alpha +1}\;{ℑ}_{\kappa_{1}+}^{\alpha }\;ϝ\left(m\left(\frac{2\kappa_{1}+\kappa_{2}}{3}\right)\right)+A_{2}^{\alpha +1}\;{ℑ}_{\frac{2\kappa_{1}+\kappa_{2}}{3}+}^{\alpha }\;ϝ\left(m\left(\frac{\kappa_{1}+2\kappa_{2}}{3}\right)\right)\\ & +A_{3}^{\alpha +1}\;{ℑ}_{\frac{\kappa_{1}+2\kappa_{2}}{3}+}^{\alpha }\;ϝ\left(m\kappa_{2}\right)]-\frac{1}{8}[3A_{1}ϝ(\kappa_{1})+5A_{1}ϝ\left(m\left(\frac{2\kappa_{1}+\kappa_{2}}{3}\right)\right)+4A_{2}ϝ\left(m\left(\frac{2\kappa_{1}+\kappa_{2}}{3}\right)\right)\\ & +4A_{2}ϝ\left(m\left(\frac{\kappa_{1}+2\kappa_{2}}{3}\right)\right)+5A_{3}ϝ\left(\frac{\kappa_{1}+2\kappa_{2}}{3}\right)+3A_{3}ϝ(m\kappa_{2})]|\\ & \leq \frac{1}{3^{p}(p)}[{\left(\int_{0}^{1}{\left|\vartheta^{\alpha }-\frac{5}{8}\right|}^{t}d\vartheta \right)}^{\frac{1}{t}}{\left(C_{1}{\left|{ϝ}^{{\;}^{\prime }}(\kappa_{1})\right|}^{q}+mC_{2}{\left|{ϝ}^{{\;}^{\prime }}\left(\frac{2\kappa_{1}+\kappa_{2}}{3}\right)\right|}^{q}\right)}^{\frac{1}{q}}\\ & +{\left(\int_{0}^{1}{\left|\vartheta^{\alpha }-\frac{1}{2}\right|}^{t}d\vartheta \right)}^{\frac{1}{t}}{\left(C_{3}{\left|{ϝ}^{{\;}^{\prime }}\left(\frac{2\kappa_{1}+\kappa_{2}}{3}\right)\right|}^{q}+mC_{4}{\left|{ϝ}^{{\;}^{\prime }}\left(\frac{\kappa_{1}+2\kappa_{2}}{3}\right)\right|}^{q}\right)}^{\frac{1}{q}}\\ & +{\left(\int_{0}^{1}{\left|\vartheta^{\alpha }-\frac{3}{8}\right|}^{t}d\vartheta \right)}^{\frac{1}{t}}(C_{5}{\left|{ϝ}^{{\;}^{\prime }}\left(\frac{\kappa_{1}+2\kappa_{2}}{3}\right)\right|}^{q}+mC_{6}{\left|{ϝ}^{{\;}^{\prime }}(\kappa_{2})\right|}^{q}{)}^{\frac{1}{q}}], \end{array}$$ (8)

*where A*_*1*_*, A*_*2*_*, and A*_*3*_ *are given in (2), and*


$$\begin{array}{cc} & C_{1}=\int_{0}^{1}\frac{\sinh\left[p\left(1-s(1-\vartheta )\right)\left(\frac{k_{2}-k_{1}}{3}\right)\right]}{\sinh\left[p\left(\frac{k_{2}-k_{1}}{3}\right)\right]{\left[\vartheta (\kappa_{1}{)}^{p}+m(1-\vartheta )(\frac{2\kappa_{1}+\kappa_{2}}{3}{)}^{p}\right]}^{q\left(1-\frac{1}{p}\right)}}d\vartheta ,\\ & C_{2}=\int_{0}^{1}\frac{\sinh\left[p\left(1-s\vartheta \right)\left(\frac{k_{2}-k_{1}}{3}\right)\right]}{\sinh\left[p\left(\frac{k_{2}-k_{1}}{3}\right)\right]{\left[\vartheta (\kappa_{1}{)}^{p}+m(1-\vartheta )(\frac{2\kappa_{1}+\kappa_{2}}{3}{)}^{p}\right]}^{q\left(1-\frac{1}{p}\right)}}d\vartheta ,\\ & C_{3}=\int_{0}^{1}\frac{\sinh\left[p\left(1-s(1-\vartheta )\right)\left(\frac{k_{2}-k_{1}}{3}\right)\right]}{\sinh\left[p\left(\frac{k_{2}-k_{1}}{3}\right)\right]{\left[\vartheta (\frac{2\kappa_{1}+\kappa_{2}}{3}{)}^{p}+m(1-\vartheta )(\frac{\kappa_{1}+2\kappa_{2}}{3}{)}^{p}\right]}^{q\left(1-\frac{1}{p}\right)}}d\vartheta ,\\ & C_{4}=\int_{0}^{1}\frac{\sinh\left[p\left(1-s\vartheta \right)\left(\frac{k_{2}-k_{1}}{3}\right)\right]}{\sinh\left[p\left(\frac{k_{2}-k_{1}}{3}\right)\right]{\left[\vartheta (\frac{2\kappa_{1}+\kappa_{2}}{3}{)}^{p}+m(1-\vartheta )(\frac{\kappa_{1}+2\kappa_{2}}{3}{)}^{p}\right]}^{q\left(1-\frac{1}{p}\right)}}d\vartheta ,\\ & C_{5}=\int_{0}^{1}\frac{\sinh\left[p\left(1-s(1-\vartheta )\right)\left(\frac{k_{2}-k_{1}}{3}\right)\right]}{\sinh\left[p\left(\frac{k_{2}-k_{1}}{3}\right)\right]{\left[\vartheta (\frac{\kappa_{1}+2\kappa_{2}}{3}{)}^{p}+m(1-\vartheta )\kappa_{2}^{p}\right]}^{q\left(1-\frac{1}{p}\right)}}d\vartheta ,\\ & C_{6}=\int_{0}^{1}\frac{\sinh\left[p\left(1-s\vartheta \right)\left(\frac{k_{2}-k_{1}}{3}\right)\right]}{\sinh\left[p\left(\frac{k_{2}-k_{1}}{3}\right)\right]{\left[\vartheta (\frac{\kappa_{1}+2\kappa_{2}}{3}{)}^{p}+m(1-\vartheta )\kappa_{2}^{p}\right]}^{q\left(1-\frac{1}{p}\right)}}d\vartheta . \end{array}$$

*Proof.* By using the property of absolute value on (3) and applying Hölder’s inequality


$$\begin{array}{cc} & |(3{)}^{p(\alpha -1)}\;\Gamma (\alpha +1)[A_{1}^{\alpha +1}\;{ℑ}_{\kappa_{1}+}^{\alpha }\;ϝ\left(m\left(\frac{2\kappa_{1}+\kappa_{2}}{3}\right)\right)+A_{2}^{\alpha +1}\;{ℑ}_{\frac{2\kappa_{1}+\kappa_{2}}{3}+}^{\alpha }\;ϝ\left(m\left(\frac{\kappa_{1}+2\kappa_{2}}{3}\right)\right)\\ & +A_{3}^{\alpha +1}\;{ℑ}_{\frac{\kappa_{1}+2\kappa_{2}}{3}+}^{\alpha }\;ϝ\left(m\kappa_{2}\right)]-\frac{1}{8}[3A_{1}ϝ(\kappa_{1})+5A_{1}ϝ\left(m\left(\frac{2\kappa_{1}+\kappa_{2}}{3}\right)\right)+4A_{2}ϝ\left(m\left(\frac{2\kappa_{1}+\kappa_{2}}{3}\right)\right)\\ & +4A_{2}ϝ\left(m\left(\frac{\kappa_{1}+2\kappa_{2}}{3}\right)\right)+5A_{3}ϝ\left(\frac{\kappa_{1}+2\kappa_{2}}{3}\right)+3A_{3}ϝ(m\kappa_{2})]|\\ & \leq \frac{1}{3^{p}(p)}[\int_{0}^{1}\left|\vartheta^{\alpha }-\frac{5}{8}\right|\frac{\left|{ϝ}^{{\;}^{\prime }}\left({\left[\vartheta (\kappa_{1}{)}^{p}+m(1-\vartheta ){\left(\frac{2\kappa_{1}+\kappa_{2}}{3}\right)}^{p}\right]}^{\frac{1}{p}}\right)\right|d\vartheta }{{\left[\vartheta (\kappa_{1}{)}^{p}+m(1-\vartheta )(\frac{2\kappa_{1}+\kappa_{2}}{3}{)}^{p}\right]}^{1-\frac{1}{p}}}\\ & +\int_{0}^{1}\left|\vartheta^{\alpha }-\frac{1}{2}\right|\frac{\left|{ϝ}^{{\;}^{\prime }}\left({\left[\vartheta {\left(\frac{2\kappa_{1}+\kappa_{2}}{3}\right)}^{p}+m(1-\vartheta ){\left(\frac{\kappa_{1}+2\kappa_{2}}{3}\right)}^{p}\right]}^{\frac{1}{p}}\right)\right|}{{\left[\vartheta (\frac{2\kappa_{1}+\kappa_{2}}{3}{)}^{p}+m(1-\vartheta )(\frac{\kappa_{1}+2\kappa_{2}}{3}{)}^{p}\right]}^{1-\frac{1}{p}}}d\vartheta \\ & +\int_{0}^{1}\left|\vartheta^{\alpha }-\frac{3}{8}\right|\frac{\left|{ϝ}^{{\;}^{\prime }}\left({\left[\vartheta {\left(\frac{\kappa_{1}+2\kappa_{2}}{3}\right)}^{p}+m(1-\vartheta )\kappa_{2}^{p}\right]}^{\frac{1}{p}}\right)\right|}{{\left[\vartheta (\frac{\kappa_{1}+2\kappa_{2}}{3}{)}^{p}+m(1-\vartheta )\kappa_{2}^{p}\right]}^{1-\frac{1}{p}}}d\vartheta ] \end{array}$$

$$\begin{array}{cc} & \leq \frac{1}{3^{p}(p)}[{\left(\int_{0}^{1}{\left|\vartheta^{\alpha }-\frac{5}{8}\right|}^{t}d\vartheta \right)}^{\frac{1}{t}}{\left(\int_{0}^{1}\frac{{\left|{ϝ}^{{\;}^{\prime }}\left({\left[\vartheta (\kappa_{1}{)}^{p}+m(1-\vartheta ){\left(\frac{2\kappa_{1}+\kappa_{2}}{3}\right)}^{p}\right]}^{\frac{1}{p}}\right)\right|}^{q}d\vartheta }{{\left[\vartheta (\kappa_{1}{)}^{p}+m(1-\vartheta )(\frac{2\kappa_{1}+\kappa_{2}}{3}{)}^{p}\right]}^{q\left(1-\frac{1}{p}\right)}}\right)}^{\frac{1}{q}}\\ & +{\left(\int_{0}^{1}{\left|\vartheta^{\alpha }-\frac{1}{2}\right|}^{t}d\vartheta \right)}^{\frac{1}{t}}{\left(\int_{0}^{1}\frac{{\left|{ϝ}^{{\;}^{\prime }}\left({\left[\vartheta {\left(\frac{2\kappa_{1}+\kappa_{2}}{3}\right)}^{p}+m(1-\vartheta ){\left(\frac{\kappa_{1}+2\kappa_{2}}{3}\right)}^{p}\right]}^{\frac{1}{p}}\right)\right|}^{q}d\vartheta }{{\left[\vartheta {\left(\frac{2\kappa_{1}+\kappa_{2}}{3}\right)}^{p}+m(1-\vartheta )(\frac{2\kappa_{1}+\kappa_{2}}{3}{)}^{p}\right]}^{q\left(1-\frac{1}{p}\right)}}\right)}^{\frac{1}{q}}\\ & +{\left(\int_{0}^{1}{\left|\vartheta^{\alpha }-\frac{3}{8}\right|}^{t}d\vartheta \right)}^{\frac{1}{t}}{\left(\int_{0}^{1}\frac{{\left|{ϝ}^{{\;}^{\prime }}\left({\left[\vartheta {\left(\frac{\kappa_{1}+2\kappa_{2}}{3}\right)}^{p}+m(1-\vartheta )\kappa_{2}^{p}\right]}^{\frac{1}{p}}\right)\right|}^{q}d\vartheta }{{\left[\vartheta {\left(\frac{\kappa_{1}+2\kappa_{2}}{3}\right)}^{p}+m(1-\vartheta )\kappa_{2}^{p}\right]}^{q\left(1-\frac{1}{p}\right)}}\right)}^{\frac{1}{q}}]. \end{array}$$

By utilizing modified hyperbolic *p*-convexity of $|{ϝ}^{{\;}^{\prime }}{|}^{q}$,


$$\begin{array}{cc} & |(3{)}^{p(\alpha -1)}\;\Gamma (\alpha +1)[A_{1}^{\alpha +1}\;{ℑ}_{\kappa_{1}+}^{\alpha }\;ϝ\left(m\left(\frac{2\kappa_{1}+\kappa_{2}}{3}\right)\right)+A_{2}^{\alpha +1}\;{ℑ}_{\frac{2\kappa_{1}+\kappa_{2}}{3}+}^{\alpha }\;ϝ\left(m\left(\frac{\kappa_{1}+2\kappa_{2}}{3}\right)\right)\\ & +A_{3}^{\alpha +1}\;{ℑ}_{\frac{\kappa_{1}+2\kappa_{2}}{3}+}^{\alpha }\;ϝ\left(m\kappa_{2}\right)]-\frac{1}{8}[3A_{1}ϝ(\kappa_{1})+5A_{1}ϝ\left(m\left(\frac{2\kappa_{1}+\kappa_{2}}{3}\right)\right)+4A_{2}ϝ\left(m\left(\frac{2\kappa_{1}+\kappa_{2}}{3}\right)\right)\\ & +4A_{2}ϝ\left(m\left(\frac{\kappa_{1}+2\kappa_{2}}{3}\right)\right)+5A_{3}ϝ\left(\frac{\kappa_{1}+2\kappa_{2}}{3}\right)+3A_{3}ϝ(m\kappa_{2})]| \end{array}$$

$$\begin{array}{cc} | & \leq \frac{1}{3^{p}(p)}[{\left(\int_{0}^{1}{\left|\vartheta^{\alpha }-\frac{5}{8}\right|}^{t}d\vartheta \right)}^{\frac{1}{t}}\\ & \;\times ({\left|{ϝ}^{{\;}^{\prime }}(\kappa_{1})\right|}^{q}\int_{0}^{1}\frac{\sinh\left[p\left(1-s(1-\vartheta )\right)\left(\frac{k_{2}-k_{1}}{3}\right)\right]}{\sinh\left[p\left(\frac{k_{2}-k_{1}}{3}\right)\right]{\left[\vartheta \kappa_{1}^{p}+m(1-\vartheta ){\left(\frac{2\kappa_{1}+\kappa_{2}}{3}\right)}^{p}\right]}^{q\left(1-\frac{1}{p}\right)}}d\vartheta \\ & \;+m{\left|{ϝ}^{{\;}^{\prime }}\left(\frac{2\kappa_{1}+\kappa_{2}}{3}\right)\right|}^{q}\int_{0}^{1}\frac{\sinh\left[p\left(1-s\vartheta \right)\left(\frac{k_{2}-k_{1}}{3}\right)\right]}{\sinh\left[p\left(\frac{k_{2}-k_{1}}{3}\right)\right]{\left[\vartheta \kappa_{1}^{p}+m(1-\vartheta ){\left(\frac{2\kappa_{1}+\kappa_{2}}{3}\right)}^{p}\right]}^{q\left(1-\frac{1}{p}\right)}}d\vartheta {)}^{\frac{1}{q}}\\ & \;+{\left(\int_{0}^{1}{\left|\vartheta^{\alpha }-\frac{1}{2}\right|}^{t}d\vartheta \right)}^{\frac{1}{t}}\\ & \;\times ({\left|{ϝ}^{{\;}^{\prime }}\left(\frac{2\kappa_{1}+\kappa_{2}}{3}\right)\right|}^{q}\int_{0}^{1}\frac{\sinh\left[p\left(1-s(1-\vartheta )\right)\left(\frac{k_{2}-k_{1}}{3}\right)\right]}{\sinh\left[p\left(\frac{k_{2}-k_{1}}{3}\right)\right]{\left[\vartheta {\left(\frac{2\kappa_{1}+\kappa_{2}}{3}\right)}^{p}+m(1-\vartheta ){\left(\frac{\kappa_{1}+2\kappa_{2}}{3}\right)}^{p}\right]}^{q\left(1-\frac{1}{p}\right)}}d\vartheta \\ & \;+m{\left|{ϝ}^{{\;}^{\prime }}\left(\frac{\kappa_{1}+2\kappa_{2}}{3}\right)\right|}^{q}\int_{0}^{1}\frac{\sinh\left[p\left(1-s\vartheta \right)\left(\frac{k_{2}-k_{1}}{3}\right)\right]}{\sinh\left[p\left(\frac{k_{2}-k_{1}}{3}\right)\right]{\left[\vartheta {\left(\frac{2\kappa_{1}+\kappa_{2}}{3}\right)}^{p}+m(1-\vartheta ){\left(\frac{\kappa_{1}+2\kappa_{2}}{3}\right)}^{p}\right]}^{q\left(1-\frac{1}{p}\right)}}d\vartheta {)}^{\frac{1}{q}}\\ & \;+{\left(\int_{0}^{1}{\left|\vartheta^{\alpha }-\frac{3}{8}\right|}^{t}d\vartheta \right)}^{\frac{1}{t}}\\ & \;\times ({\left|{ϝ}^{{\;}^{\prime }}\left(\frac{\kappa_{1}+2\kappa_{2}}{3}\right)\right|}^{q}\int_{0}^{1}\frac{\sinh\left[p\left(1-s(1-\vartheta )\right)\left(\frac{k_{2}-k_{1}}{3}\right)\right]}{\sinh\left[p\left(\frac{k_{2}-k_{1}}{3}\right)\right]{\left[\vartheta {\left(\frac{\kappa_{1}+2\kappa_{2}}{3}\right)}^{p}+m(1-\vartheta )\kappa_{2}^{p}\right]}^{q\left(1-\frac{1}{p}\right)}}d\vartheta \\ & \;+m{\left|{ϝ}^{{\;}^{\prime }}\left(\kappa_{2}\right)\right|}^{q}\int_{0}^{1}\frac{\sinh\left[p\left(1-s\vartheta \right)\left(\frac{k_{2}-k_{1}}{3}\right)\right]}{\sinh\left[p\left(\frac{k_{2}-k_{1}}{3}\right)\right]{\left[\vartheta {\left(\frac{\kappa_{1}+2\kappa_{2}}{3}\right)}^{p}+m(1-\vartheta )\kappa_{2}^{p}\right]}^{q\left(1-\frac{1}{p}\right)}}d\vartheta {)}^{\frac{1}{q}}], \end{array}$$

which is the required proof. □

**Remark 3.8.** *(a) By substituting p = 1, s = 1, m = 1, and* sinh(p𝔱)=p𝔱 *in (8), it reduces to Theorem 6 of [25].*

*(b) If, in addition, we set* α=1*, p = 1, s = 1, m = 1, and* sinh(p𝔱)=p𝔱*, then it further reduces to the corresponding Remark 5 of [25].*

**Example 3.9.** *Define a function* ϝ:[0,1]→ℝ *by* $ϝ(\vartheta )=\frac{\vartheta^{3}}{3}$  *such that* ${ϝ}^{{\;}^{\prime }}(\vartheta )=\vartheta^{2}$*, and* $\left|{ϝ}^{{\;}^{\prime }}\right|$ *is modified hyperbolic p-convex on [0,1]. By taking* $\kappa_{2}=1,\kappa_{1}=0,m=0.6,s=0.5,q=2,p=2$*, and* α=0.2*, under these assumptions, inequality (8) reduces to* 0.0344≤0.21101.

*Also it is clear from* Figs 3 *and* 4 *that* LHS≤RHS *for all* α>0.

**Fig 3:**
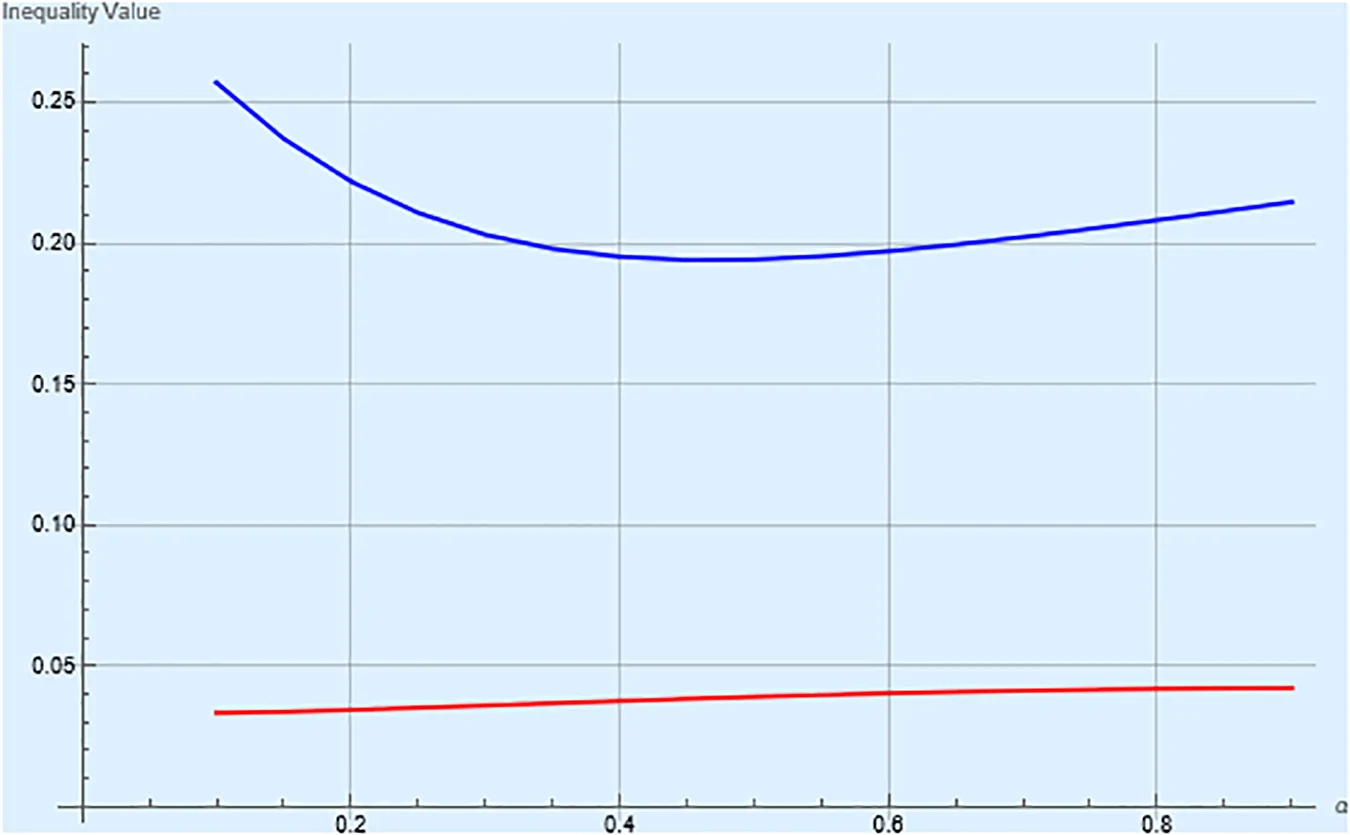
2D graphical representation of Example 3.9 for inequality (8) with $\kappa_{2}=1,\kappa_{1}=0,m=0.6,s=0.5,q=2,p=2$. Horizontal axis: α, vertical axis: Inequality value. Blue curve: LHS, red curve: RHS.

**Fig 4:**
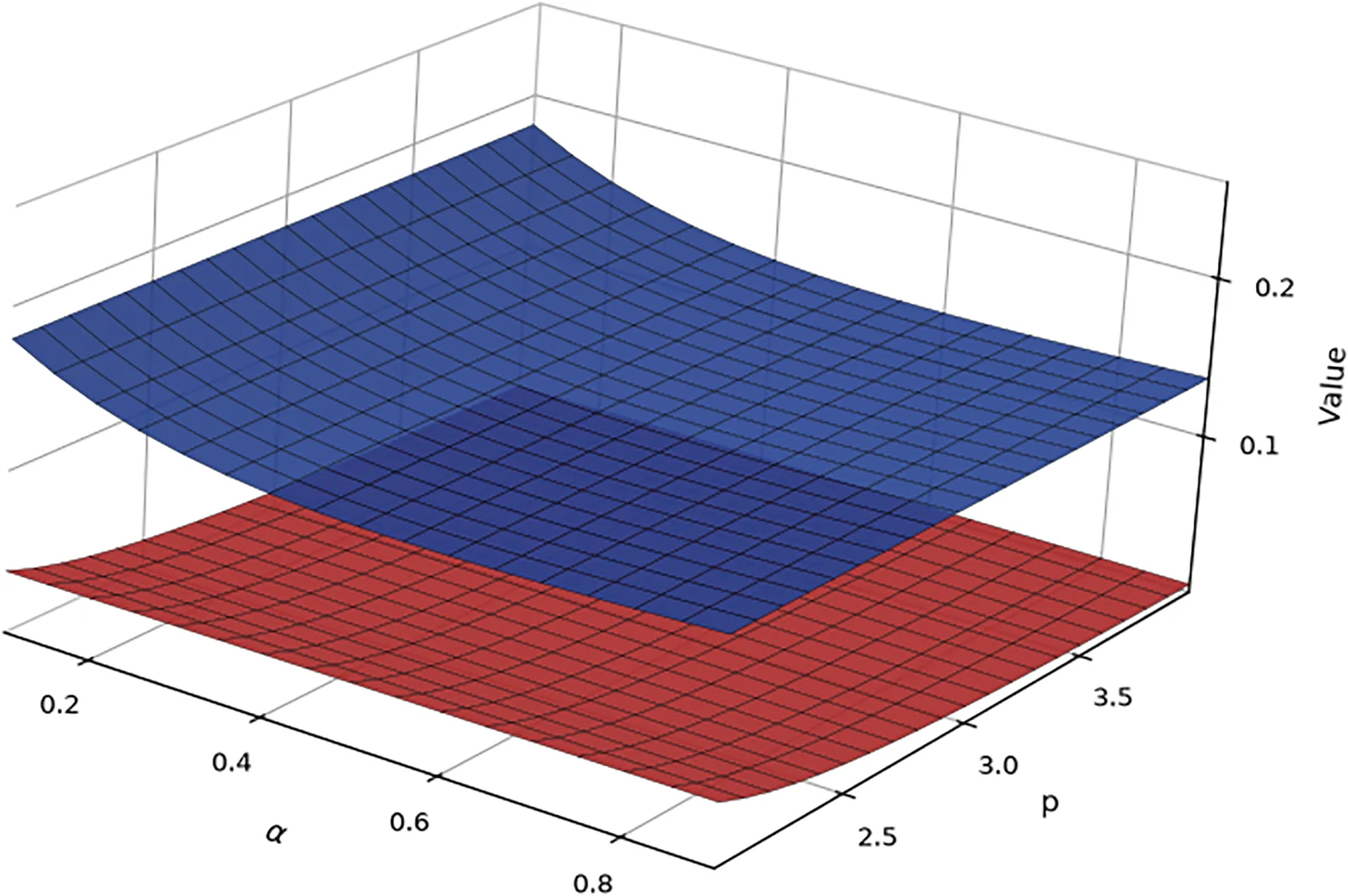
3D graphical representation of Example 3.9. Blue surface: LHS, red surface: RHS as functions of α (horizontal) and *p* (depth). The inequality holds for all shown values.

**Theorem 3.10.** *Assume the conditions of Lemma 3.1 are satisfied. If* $|{ϝ}^{\prime }{|}^{q}$*,* q≥1*, is a modified hyperbolic p-convex function, then the subsequent Newton-type inequality follows*


$$\begin{array}{cc} & |(3{)}^{p(\alpha -1)}\;\Gamma (\alpha +1)[A_{1}^{\alpha +1}\;{ℑ}_{\kappa_{1}+}^{\alpha }\;ϝ\left(m\left(\frac{2\kappa_{1}+\kappa_{2}}{3}\right)\right)+A_{2}^{\alpha +1}\;{ℑ}_{\frac{2\kappa_{1}+\kappa_{2}}{3}+}^{\alpha }\;ϝ\left(m\left(\frac{\kappa_{1}+2\kappa_{2}}{3}\right)\right)\\ & +A_{3}^{\alpha +1}\;{ℑ}_{\frac{\kappa_{1}+2\kappa_{2}}{3}+}^{\alpha }\;ϝ\left(m\kappa_{2}\right)]-\frac{1}{8}[3A_{1}ϝ(\kappa_{1})+5A_{1}ϝ\left(m\left(\frac{2\kappa_{1}+\kappa_{2}}{3}\right)\right)+4A_{2}ϝ\left(m\left(\frac{2\kappa_{1}+\kappa_{2}}{3}\right)\right)\\ & +4A_{2}ϝ\left(m\left(\frac{\kappa_{1}+2\kappa_{2}}{3}\right)\right)+5A_{3}ϝ\left(\frac{\kappa_{1}+2\kappa_{2}}{3}\right)+3A_{3}ϝ(m\kappa_{2})]| \end{array}$$ (9)

$$\begin{array}{cc} & \leq \frac{1}{3^{p}(p)}[D_{1}^{1-\frac{1}{q}}{\left(D_{2}{\left|{ϝ}^{{\;}^{\prime }}(\kappa_{1})\right|}^{q}+mD_{3}{\left|{ϝ}^{{\;}^{\prime }}\left(\frac{2\kappa_{1}+\kappa_{2}}{3}\right)\right|}^{q}\right)}^{\frac{1}{q}}\\ & +D_{4}^{1-\frac{1}{q}}{\left(D_{5}{\left|{ϝ}^{{\;}^{\prime }}\left(\frac{2\kappa_{1}+\kappa_{2}}{3}\right)\right|}^{q}+mD_{6}{\left|{ϝ}^{{\;}^{\prime }}\left(\frac{\kappa_{1}+2\kappa_{2}}{3}\right)\right|}^{q}\right)}^{\frac{1}{q}}\\ & +D_{7}^{1-\frac{1}{q}}{\left(D_{8}{\left|{ϝ}^{{\;}^{\prime }}\left(\frac{\kappa_{1}+2\kappa_{2}}{3}\right)\right|}^{q}+mD_{9}{\left|{ϝ}^{{\;}^{\prime }}(\kappa_{2})\right|}^{q}\right)}^{\frac{1}{q}}], \end{array}$$ (10)

*where A*_*1*_*, A*_*2*_*, and A*_*3*_ *are given in (2), and*


$$\begin{array}{cc} & D_{1}=\int_{0}^{1}\frac{\left|\vartheta^{\alpha }-\frac{5}{8}\right|}{{\left[\vartheta (\kappa_{1}{)}^{p}+m(1-\vartheta )(\frac{2\kappa_{1}+\kappa_{2}}{3}{)}^{p}\right]}^{\left(1-\frac{1}{p}\right)}}d\vartheta ,\\ & D_{2}=\int_{0}^{1}\frac{\sinh\left[p\left(1-s(1-\vartheta )\right)\left(\frac{k_{2}-k_{1}}{3}\right)\right]\left|\vartheta^{\alpha }-\frac{5}{8}\right|}{\sinh\left[p\left(\frac{k_{2}-k_{1}}{3}\right)\right]{\left[\vartheta (\kappa_{1}{)}^{p}+m(1-\vartheta )(\frac{2\kappa_{1}+\kappa_{2}}{3}{)}^{p}\right]}^{\left(1-\frac{1}{p}\right)}}d\vartheta ,\\ & D_{3}=\int_{0}^{1}\frac{\sinh\left[p\left(1-s\vartheta \right)\left(\frac{k_{2}-k_{1}}{3}\right)\right]\left|\vartheta^{\alpha }-\frac{5}{8}\right|}{\sinh\left[p\left(\frac{k_{2}-k_{1}}{3}\right)\right]{\left[\vartheta (\kappa_{1}{)}^{p}+m(1-\vartheta )(\frac{2\kappa_{1}+\kappa_{2}}{3}{)}^{p}\right]}^{\left(1-\frac{1}{p}\right)}}d\vartheta ,\\ & D_{4}=\int_{0}^{1}\frac{\left|\vartheta^{\alpha }-\frac{1}{2}\right|}{{\left[\vartheta (\frac{2\kappa_{1}+\kappa_{2}}{3}{)}^{p}+m(1-\vartheta )(\frac{\kappa_{1}+2\kappa_{2}}{3}{)}^{p}\right]}^{\left(1-\frac{1}{p}\right)}}d\vartheta ,\\ & D_{5}=\int_{0}^{1}\frac{\sinh\left[p\left(1-s(1-\vartheta )\right)\left(\frac{k_{2}-k_{1}}{3}\right)\right]\left|\vartheta^{\alpha }-\frac{1}{2}\right|}{\sinh\left[p\left(\frac{k_{2}-k_{1}}{3}\right)\right]{\left[\vartheta (\frac{2\kappa_{1}+\kappa_{2}}{3}{)}^{p}+m(1-\vartheta )(\frac{\kappa_{1}+2\kappa_{2}}{3}{)}^{p}\right]}^{\left(1-\frac{1}{p}\right)}}d\vartheta ,\\ & D_{6}=\int_{0}^{1}\frac{\sinh\left[p\left(1-s\vartheta \right)\left(\frac{k_{2}-k_{1}}{3}\right)\right]\left|\vartheta^{\alpha }-\frac{1}{2}\right|}{\sinh\left[p\left(\frac{k_{2}-k_{1}}{3}\right)\right]{\left[\vartheta (\frac{2\kappa_{1}+\kappa_{2}}{3}{)}^{p}+m(1-\vartheta )(\frac{\kappa_{1}+2\kappa_{2}}{3}{)}^{p}\right]}^{\left(1-\frac{1}{p}\right)}}d\vartheta ,\\ & D_{7}=\int_{0}^{1}\frac{\left|\vartheta^{\alpha }-\frac{3}{8}\right|}{{\left[\vartheta (\frac{\kappa_{1}+2\kappa_{2}}{3}{)}^{p}+m(1-\vartheta )\kappa_{2}^{p}\right]}^{\left(1-\frac{1}{p}\right)}}d\vartheta ,\\ & D_{8}=\int_{0}^{1}\frac{\sinh\left[p\left(1-s(1-\vartheta )\right)\left(\frac{k_{2}-k_{1}}{3}\right)\right]\left|\vartheta^{\alpha }-\frac{3}{8}\right|}{\sinh\left[p\left(\frac{k_{2}-k_{1}}{3}\right)\right]{\left[\vartheta (\frac{\kappa_{1}+2\kappa_{2}}{3}{)}^{p}+m(1-\vartheta )\kappa_{2}^{p}\right]}^{\left(1-\frac{1}{p}\right)}}d\vartheta ,\\ & D_{9}=\int_{0}^{1}\frac{\sinh\left[p\left(1-s\vartheta \right)\left(\frac{k_{2}-k_{1}}{3}\right)\right]\left|\vartheta^{\alpha }-\frac{3}{8}\right|}{\sinh\left[p\left(\frac{k_{2}-k_{1}}{3}\right)\right]{\left[\vartheta (\frac{\kappa_{1}+2\kappa_{2}}{3}{)}^{p}+m(1-\vartheta )\kappa_{2}^{p}\right]}^{\left(1-\frac{1}{p}\right)}}d\vartheta . \end{array}$$

*Proof.* By using the property of absolute value on (3) and applying power-mean inequality


$$\begin{array}{cc} & |(3{)}^{p(\alpha -1)}\;\Gamma (\alpha +1)[A_{1}^{\alpha +1}\;{ℑ}_{\kappa_{1}+}^{\alpha }\;ϝ\left(m\left(\frac{2\kappa_{1}+\kappa_{2}}{3}\right)\right)+A_{2}^{\alpha +1}\;{ℑ}_{\frac{2\kappa_{1}+\kappa_{2}}{3}+}^{\alpha }\;ϝ\left(m\left(\frac{\kappa_{1}+2\kappa_{2}}{3}\right)\right)\\ & +A_{3}^{\alpha +1}\;{ℑ}_{\frac{\kappa_{1}+2\kappa_{2}}{3}+}^{\alpha }\;ϝ\left(m\kappa_{2}\right)]-\frac{1}{8}[3A_{1}ϝ(\kappa_{1})+5A_{1}ϝ\left(m\left(\frac{2\kappa_{1}+\kappa_{2}}{3}\right)\right)+4A_{2}ϝ\left(m\left(\frac{2\kappa_{1}+\kappa_{2}}{3}\right)\right)\\ & +4A_{2}ϝ\left(m\left(\frac{\kappa_{1}+2\kappa_{2}}{3}\right)\right)+5A_{3}ϝ\left(\frac{\kappa_{1}+2\kappa_{2}}{3}\right)+3A_{3}ϝ(m\kappa_{2})]| \end{array}$$

$$\begin{array}{cc} & \leq \frac{1}{3^{p}(p)}[\int_{0}^{1}\left|\vartheta^{\alpha }-\frac{5}{8}\right|\frac{\left|{ϝ}^{{\;}^{\prime }}\left({\left[\vartheta \kappa_{1}^{p}+m(1-\vartheta ){\left(\frac{2\kappa_{1}+\kappa_{2}}{3}\right)}^{p}\right]}^{\frac{1}{p}}\right)\right|d\vartheta }{{\left[\vartheta \kappa_{1}^{p}+m(1-\vartheta )(\frac{2\kappa_{1}+\kappa_{2}}{3}{)}^{p}\right]}^{1-\frac{1}{p}}}\\ & +\int_{0}^{1}\left|\vartheta^{\alpha }-\frac{1}{2}\right|\frac{\left|{ϝ}^{{\;}^{\prime }}\left({\left[\vartheta {\left(\frac{2\kappa_{1}+\kappa_{2}}{3}\right)}^{p}+m(1-\vartheta ){\left(\frac{\kappa_{1}+2\kappa_{2}}{3}\right)}^{p}\right]}^{\frac{1}{p}}\right)\right|}{{\left[\vartheta (\frac{2\kappa_{1}+\kappa_{2}}{3}{)}^{p}+m(1-\vartheta ){\left(\frac{\kappa_{1}+2\kappa_{2}}{3}\right)}^{p}\right]}^{1-\frac{1}{p}}}d\vartheta \\ & +\int_{0}^{1}\left|\vartheta^{\alpha }-\frac{3}{8}\right|\frac{\left|{ϝ}^{{\;}^{\prime }}\left({\left[\vartheta {\left(\frac{\kappa_{1}+2\kappa_{2}}{3}\right)}^{p}+m(1-\vartheta )\kappa_{2}^{p}\right]}^{\frac{1}{p}}\right)\right|}{{\left[\vartheta (\frac{\kappa_{1}+2\kappa_{2}}{3}{)}^{p}+m(1-\vartheta )\kappa_{2}^{p}\right]}^{1-\frac{1}{p}}}d\vartheta ] \end{array}$$

$$\begin{array}{cc} & \leq \frac{1}{3^{p}(p)}[{\left(\int_{0}^{1}\frac{\left|\vartheta^{\alpha }-\frac{5}{8}\right|}{{\left[\vartheta \kappa_{1}^{p}+m(1-\vartheta )(\frac{2\kappa_{1}+\kappa_{2}}{3}{)}^{p}\right]}^{1-\frac{1}{p}}}d\vartheta \right)}^{1-\frac{1}{q}}\\ & \times (\int_{0}^{1}\frac{\left|\vartheta^{\alpha }-\frac{5}{8}\right|}{{\left[\vartheta \kappa_{1}^{p}+m(1-\vartheta ){\left(\frac{2\kappa_{1}+\kappa_{2}}{3}\right)}^{p}\right]}^{1-\frac{1}{p}}}\\ & {\times {\left|{ϝ}^{{\;}^{\prime }}\left({\left[\vartheta \kappa_{1}^{p}+m(1-\vartheta ){\left(\frac{2\kappa_{1}+\kappa_{2}}{3}\right)}^{p}\right]}^{\frac{1}{p}}\right)\right|}^{q}d\vartheta )}^{\frac{1}{q}}\\ & +{\left(\int_{0}^{1}\frac{\left|\vartheta^{\alpha }-\frac{1}{2}\right|}{{\left[\vartheta {\left(\frac{2\kappa_{1}+\kappa_{2}}{3}\right)}^{p}+m(1-\vartheta ){\left(\frac{\kappa_{1}+2\kappa_{2}}{3}\right)}^{p}\right]}^{1-\frac{1}{p}}}d\vartheta \right)}^{1-\frac{1}{q}}\\ & \times (\int_{0}^{1}\frac{\left|\vartheta^{\alpha }-\frac{1}{2}\right|}{{\left[\vartheta {\left(\frac{2\kappa_{1}+\kappa_{2}}{3}\right)}^{p}+m(1-\vartheta ){\left(\frac{\kappa_{1}+2\kappa_{2}}{3}\right)}^{p}\right]}^{1-\frac{1}{p}}}\\ & {\times {\left|{ϝ}^{{\;}^{\prime }}\left({\left[\vartheta {\left(\frac{2\kappa_{1}+\kappa_{2}}{3}\right)}^{p}+m(1-\vartheta ){\left(\frac{\kappa_{1}+2\kappa_{2}}{3}\right)}^{p}\right]}^{\frac{1}{p}}\right)\right|}^{q}d\vartheta )}^{\frac{1}{q}}\\ & +{\left(\int_{0}^{1}\frac{\left|\vartheta^{\alpha }-\frac{3}{8}\right|}{{\left[\vartheta {\left(\frac{\kappa_{1}+2\kappa_{2}}{3}\right)}^{p}+m(1-\vartheta )\kappa_{2}^{p}\right]}^{1-\frac{1}{p}}}d\vartheta \right)}^{1-\frac{1}{q}}\\ & \times (\int_{0}^{1}\frac{\left|\vartheta^{\alpha }-\frac{3}{8}\right|}{{\left[\vartheta {\left(\frac{\kappa_{1}+2\kappa_{2}}{3}\right)}^{p}+m(1-\vartheta )\kappa_{2}^{p}\right]}^{1-\frac{1}{p}}}\\ & {\times {\left|{ϝ}^{{\;}^{\prime }}\left({\left[\vartheta {\left(\frac{\kappa_{1}+2\kappa_{2}}{3}\right)}^{p}+m(1-\vartheta )\kappa_{2}^{p}\right]}^{\frac{1}{p}}\right)\right|}^{q}d\vartheta )}^{\frac{1}{q}}]. \end{array}$$

By using modified hyperbolic *p*-convexity of $|{ϝ}^{{\;}^{\prime }}{|}^{q}$,


$$\begin{array}{cc} & |(3{)}^{p(\alpha -1)}\;\Gamma (\alpha +1)[A_{1}^{\alpha +1}\;{ℑ}_{\kappa_{1}+}^{\alpha }\;ϝ\left(m\left(\frac{2\kappa_{1}+\kappa_{2}}{3}\right)\right)+A_{2}^{\alpha +1}\;{ℑ}_{\frac{2\kappa_{1}+\kappa_{2}}{3}+}^{\alpha }\;ϝ\left(m\left(\frac{\kappa_{1}+2\kappa_{2}}{3}\right)\right)\\ & +A_{3}^{\alpha +1}\;{ℑ}_{\frac{\kappa_{1}+2\kappa_{2}}{3}+}^{\alpha }\;ϝ\left(m\kappa_{2}\right)]-\frac{1}{8}[3A_{1}ϝ(\kappa_{1})+5A_{1}ϝ\left(m\left(\frac{2\kappa_{1}+\kappa_{2}}{3}\right)\right)+4A_{2}ϝ\left(m\left(\frac{2\kappa_{1}+\kappa_{2}}{3}\right)\right)\\ & +4A_{2}ϝ\left(m\left(\frac{\kappa_{1}+2\kappa_{2}}{3}\right)\right)+5A_{3}ϝ\left(\frac{\kappa_{1}+2\kappa_{2}}{3}\right)+3A_{3}ϝ(m\kappa_{2})]| \end{array}$$

$$\begin{array}{cc} & \leq \frac{1}{3^{p}(p)}[{\left(\int_{0}^{1}\frac{\left|\vartheta^{\alpha }-\frac{5}{8}\right|}{{\left[\vartheta \kappa_{1}^{p}+m(1-\vartheta )(\frac{2\kappa_{1}+\kappa_{2}}{3}{)}^{p}\right]}^{1-\frac{1}{p}}}d\vartheta \right)}^{1-\frac{1}{q}}\\ & \times ({\left|{ϝ}^{{\;}^{\prime }}(\kappa_{1})\right|}^{q}\int_{0}^{1}\frac{\sinh\left[p\left(1-s(1-\vartheta )\right)\left(\frac{k_{2}-k_{1}}{3}\right)\right]\left|\vartheta^{\alpha }-\frac{5}{8}\right|}{\sinh\left[p\left(\frac{k_{2}-k_{1}}{3}\right)\right]{\left[\vartheta \kappa_{1}^{p}+m(1-\vartheta ){\left(\frac{2\kappa_{1}+\kappa_{2}}{3}\right)}^{p}\right]}^{1-\frac{1}{p}}}d\vartheta \\ & {+m{\left|{ϝ}^{{\;}^{\prime }}\left(\frac{2\kappa_{1}+\kappa_{2}}{3}\right)\right|}^{q}\int_{0}^{1}\frac{\sinh\left[p\left(1-s\vartheta \right)\left(\frac{k_{2}-k_{1}}{3}\right)\right]\left|\vartheta^{\alpha }-\frac{5}{8}\right|}{\sinh\left[p\left(\frac{k_{2}-k_{1}}{3}\right)\right]{\left[\vartheta \kappa_{1}^{p}+m(1-\vartheta ){\left(\frac{2\kappa_{1}+\kappa_{2}}{3}\right)}^{p}\right]}^{1-\frac{1}{p}}}d\vartheta )}^{\frac{1}{q}}\\ & +{\left(\int_{0}^{1}\frac{\left|\vartheta^{\alpha }-\frac{1}{2}\right|}{{\left[\vartheta {\left(\frac{2\kappa_{1}+\kappa_{2}}{3}\right)}^{p}+m(1-\vartheta )(\frac{\kappa_{1}+2\kappa_{2}}{3}{)}^{p}\right]}^{1-\frac{1}{p}}}d\vartheta \right)}^{1-\frac{1}{q}}\\ & \times ({\left|{ϝ}^{{\;}^{\prime }}\left(\frac{2\kappa_{1}+\kappa_{2}}{3}\right)\right|}^{q}\int_{0}^{1}\frac{\sinh\left[p\left(1-s(1-\vartheta )\right)\left(\frac{k_{2}-k_{1}}{3}\right)\right]\left|\vartheta^{\alpha }-\frac{1}{2}\right|}{\sinh\left[p\left(\frac{k_{2}-k_{1}}{3}\right)\right]{\left[\vartheta {\left(\frac{2\kappa_{1}+\kappa_{2}}{3}\right)}^{p}+m(1-\vartheta ){\left(\frac{\kappa_{1}+2\kappa_{2}}{3}\right)}^{p}\right]}^{1-\frac{1}{p}}}d\vartheta \\ & {+m{\left|{ϝ}^{{\;}^{\prime }}\left(\frac{\kappa_{1}+2\kappa_{2}}{3}\right)\right|}^{q}\int_{0}^{1}\frac{\sinh\left[p\left(1-s\vartheta \right)\left(\frac{k_{2}-k_{1}}{3}\right)\right]\left|\vartheta^{\alpha }-\frac{1}{2}\right|}{\sinh\left[p\left(\frac{k_{2}-k_{1}}{3}\right)\right]{\left[\vartheta {\left(\frac{2\kappa_{1}+\kappa_{2}}{3}\right)}^{p}+m(1-\vartheta ){\left(\frac{\kappa_{1}+2\kappa_{2}}{3}\right)}^{p}\right]}^{1-\frac{1}{p}}}d\vartheta )}^{\frac{1}{q}}\\ & +{\left(\int_{0}^{1}\frac{\left|\vartheta^{\alpha }-\frac{3}{8}\right|}{{\left[\vartheta {\left(\frac{\kappa_{1}+2\kappa_{2}}{3}\right)}^{p}+m(1-\vartheta )\kappa_{2}^{p}\right]}^{1-\frac{1}{p}}}d\vartheta \right)}^{1-\frac{1}{q}}\\ & \times ({\left|{ϝ}^{{\;}^{\prime }}\left(\frac{\kappa_{1}+2\kappa_{2}}{3}\right)\right|}^{q}\int_{0}^{1}\frac{\sinh\left[p\left(1-s(1-\vartheta )\right)\left(\frac{k_{2}-k_{1}}{3}\right)\right]\left|\vartheta^{\alpha }-\frac{3}{8}\right|}{\sinh\left[p\left(\frac{k_{2}-k_{1}}{3}\right)\right]{\left[\vartheta {\left(\frac{\kappa_{1}+2\kappa_{2}}{3}\right)}^{p}+m(1-\vartheta )\kappa_{2}^{p}\right]}^{1-\frac{1}{p}}}d\vartheta \\ & {+m{\left|{ϝ}^{{\;}^{\prime }}(\kappa_{2})\right|}^{q}\int_{0}^{1}\frac{\sinh\left[p\left(1-s\vartheta \right)\left(\frac{k_{2}-k_{1}}{3}\right)\right]\left|\vartheta^{\alpha }-\frac{3}{8}\right|}{\sinh\left[p\left(\frac{k_{2}-k_{1}}{3}\right)\right]{\left[\vartheta {\left(\frac{\kappa_{1}+2\kappa_{2}}{3}\right)}^{p}+m(1-\vartheta )\kappa_{2}^{p}\right]}^{1-\frac{1}{p}}}d\vartheta )}^{\frac{1}{q}}], \end{array}$$

thus, the proof is complete. □

**Remark 3.11.** *(a) By substituting p = 1, s = 1, m = 1, and* sinh(p𝔱)=p𝔱 *in (9), it reduces to Theorem 5 of [25].*

*(b) If, in addition, we set* α=1*, p = 1, s = 1, m = 1, and* sinh(p𝔱)=p𝔱*, then it further reduces to Remark 4 of [25].*

**Example 3.12** *Let* $\left[\kappa_{1},\kappa_{2}]=[0,1\right]$*. Define a function* ϝ:[0,1]→ℝ *as* $ϝ(\vartheta )=\frac{\vartheta^{3}}{3}$ *such that* ${ϝ}^{{\;}^{\prime }}(\vartheta )=\vartheta^{2}$*. For the chosen parameters* $\kappa_{2}=1,\kappa_{1}=0,m=1,s=0.5,q=2,p=2$*, one can verify numerically that* $|{ϝ}^{{\;}^{\prime }}(\vartheta )|=\vartheta^{2}$ *satisfies Definition 2.2; the routine verification is omitted for brevity. Taking* α=0.6*, inequality (9) reduces to* 0.056567≤0.093212*. Also it is clear from* Figs 5 *and* 6 *that* redLHS≤blueRHS *for all* α>0*.*

**Fig 5:**
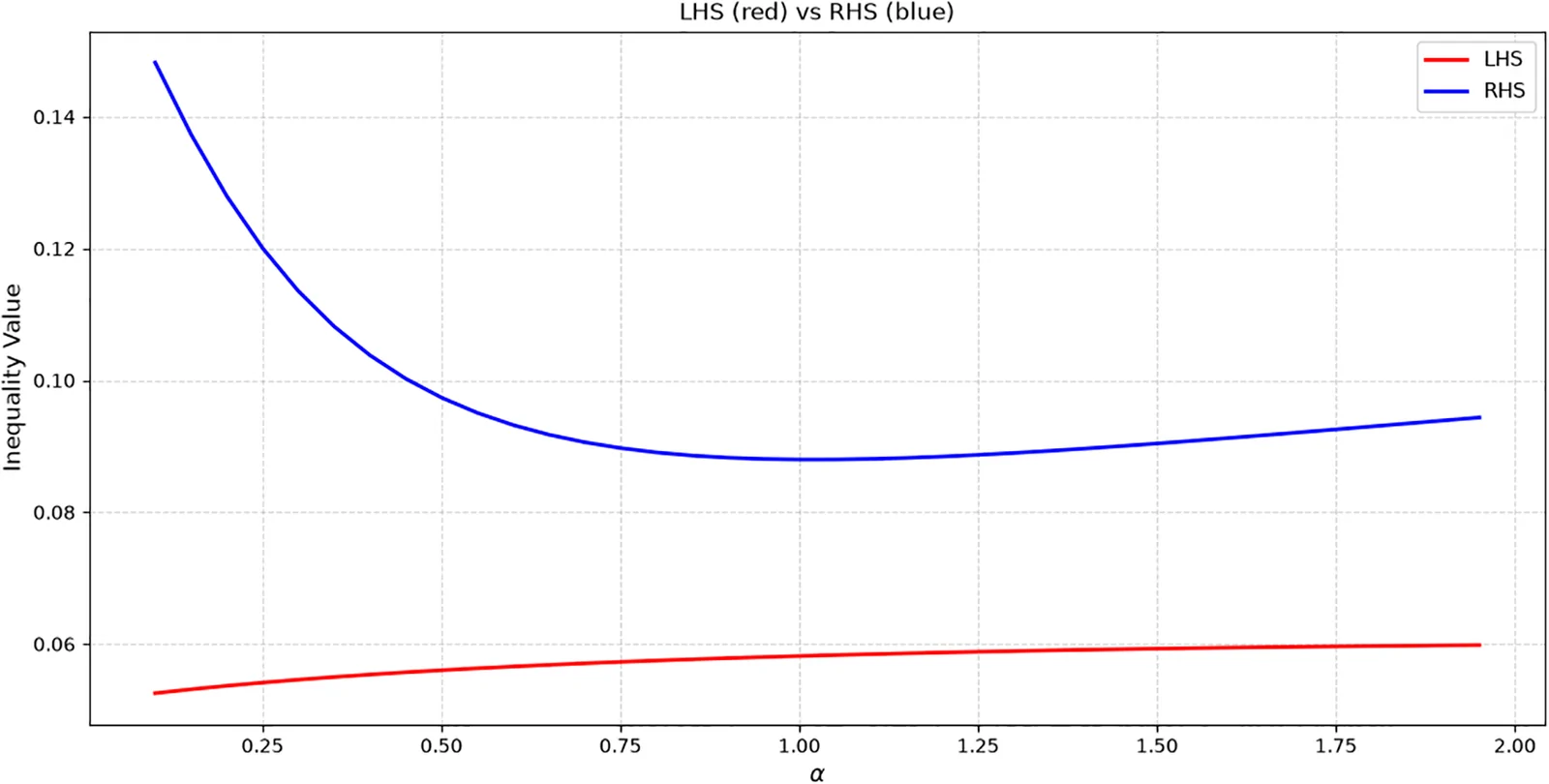
2D graphical validation of inequality (9) for Example 3.12 with $\kappa_{2}=1,\kappa_{1}=0,m=1,s=0.5,q=2,p=2$. Horizontal axis: fractional order α, vertical axis: Inequality value. Red curve: LHS, blue curve: RHS. The inequality LHS≤RHS holds for all α>0.

**Fig 6:**
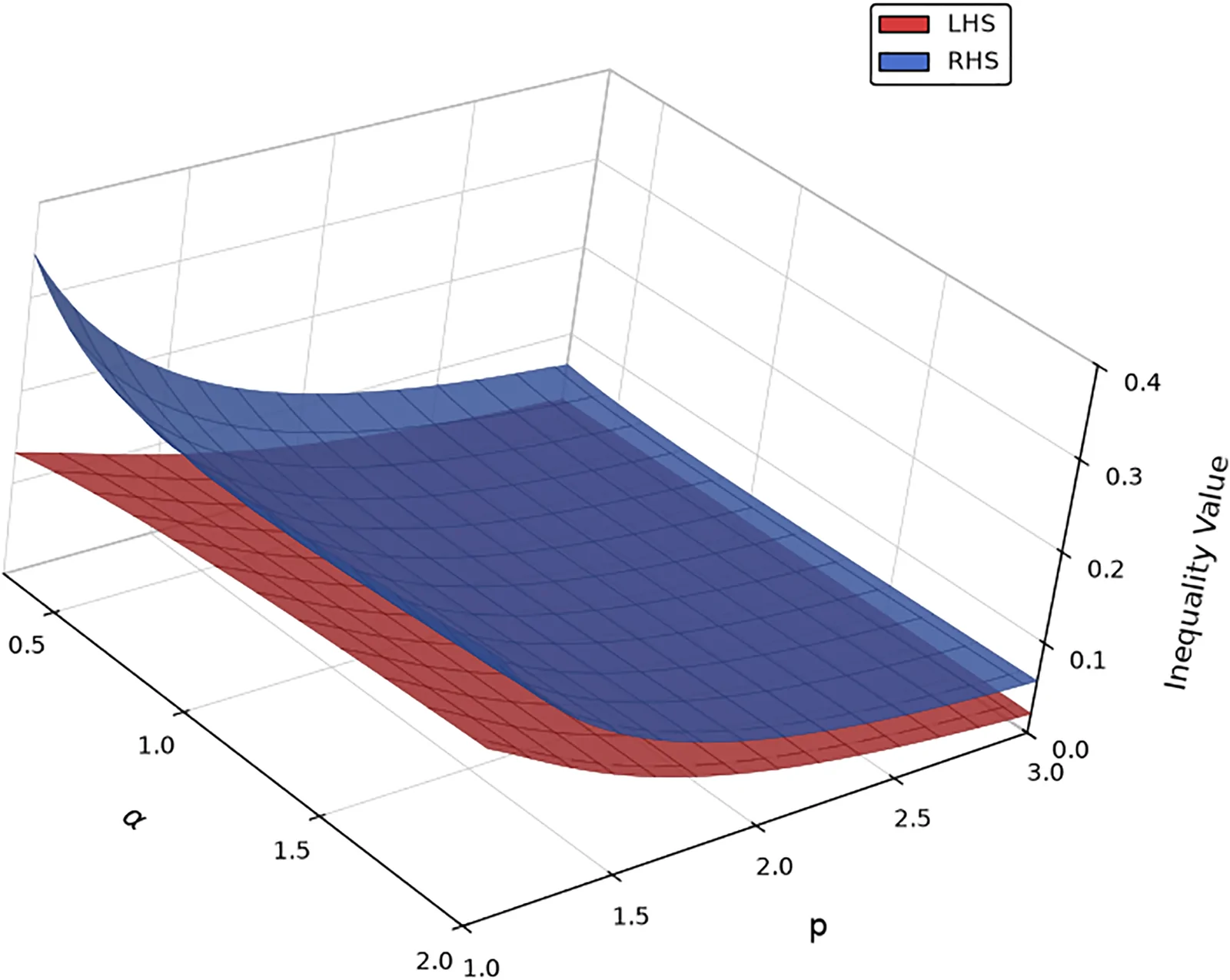
3D graphical validation of inequality (9) for Example 3.12. Blue surface: LHS, red surface: RHS as functions of α (horizontal axis) and *p* (depth axis). The red surface lies above the blue surface, confirming the inequality.

### Discussion on parameters

The parameters *m*, *s*, *p*, and α provide options to control the derived inequalities. The first parameter m∈[0,1] adjusts the weighting at the right end of an interval; the smaller *m*, the more accurate the upper/lower bound, however, narrower the set of functions. The asymmetry factor s∈[0,1] determines how much weight to assign each curvature $\kappa_{1}$ and $\kappa_{2}$. For *s* = 1, a symmetric formulation is achieved. The exponent parameter *p* > 0 defines the geometry of convexity; in particular, *p* = 1, *m* = *s* = 1, results in a conventional convexity. The fractional order α>0 controls the memory effect in the Riemann-Liouville integration operator; high values produce a smoothing effect and enlarge the upper/lower bounds.

**Comparison:**

Here, we use Lemma 3.1 to prove Theorem 3.10 and by Siththiwiratttham *et al.* [25] used Lemma 1 to prove Theorem 5 in [25]. Now, we can compare these two theorems.

By taking $\kappa_{2}=1,\kappa_{1}=0,m=1,s=0.5,q=2,p=2$, and α=0.6 in [25], inequality (12) of Theorem 5 of [25] becomes: 0.015036≤0.058312.

Putting $\kappa_{2}=1,\kappa_{1}=0,m=1,s=0.5,q=2,p=2$, and α=0.6 in our Theorem 3.10 (inequality (9)) gives: 0.056567≤0.093212.

The bound (RHS – LHS) for our inequality is 0.036645, which is less than 0.043276 from [25]. Hence (9) is more effective than inequality (12) of [25].

Table 1 shows that Theorem 3.10 yields better bounds than Theorem 5 of [25].

**Table 1:** Comparison between Theorem 3.10 and Theorem 5 of [25] for $\kappa_{2}=1$, $\kappa_{1}=0$, *m* = 1, *s* = 0.5, *q* = 2, *p* = 2.

α	*LHS* of (9)	*RHS* of (9)	*Bounds* of (9)	*LHS* of (12) of [25]	*RHS* of (12) of [25]	*Bounds* of (12) of [25]
0.6	0.056567	0.093212	0.036645	0.015036	0.058312	0.043276
0.7	0.057052	0.090599	0.033547	0.010514	0.057751	0.047236
0.8	0.057473	0.089054	0.031581	0.006562	0.057890	0.051328
1.0	0.058162	0.087977	0.029815	0.000000	0.059321	0.059321
1.2	0.058694	0.088427	0.029733	0.005211	0.061446	0.056235
1.4	0.059107	0.089645	0.030537	0.009435	0.063766	0.054331
1.6	0.059429	0.091237	0.031808	0.012920	0.066065	0.053145
1.8	0.059677	0.092996	0.033319	0.015838	0.068249	0.052411
2.0	0.059863	0.094804	0.034941	0.018313	0.070284	0.051971
2.1	0.059936	0.095705	0.035770	0.019413	0.071242	0.051829
2.2	0.059996	0.096598	0.036603	0.020435	0.072160	0.051726

Also authenticity of (9) and (12) of [25] can be checked from Fig 7. Positive difference is generated by inequality (9) is shown in red colour and positive difference generated by inequality (12) of [25] is shown in green colour respectively.

**Fig 7:**
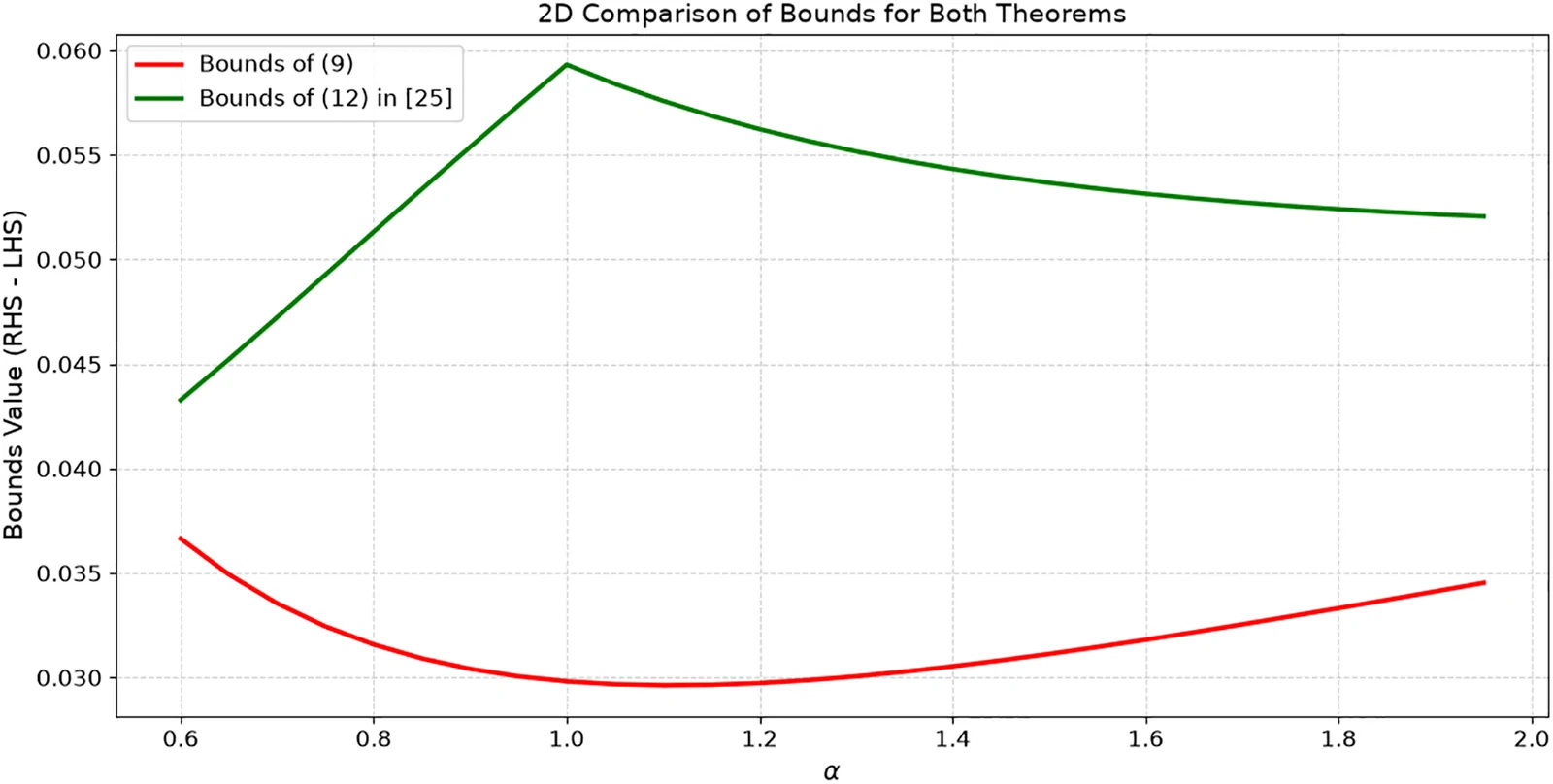
Comparison of bound differences: red curve shows RHS-LHS for our inequality (9), green curve for inequality (12) of [25]. Horizontal axis: α, vertical axis: bounds value. The red curve lies consistently below the green one, indicating tighter bounds.

Further validity of our fractional Newton-type inequalities is established through the bar chart shown in Fig 8. The positive heights of the bars indicate that RHS−LHS≥0 for all values of α, which is a prerequisite for our inequality to hold. Our observation reveals that (9) is better than (12) of [25] since the heights of the red bars are smaller than those of the green ones in Fig 8.

**Fig 8:**
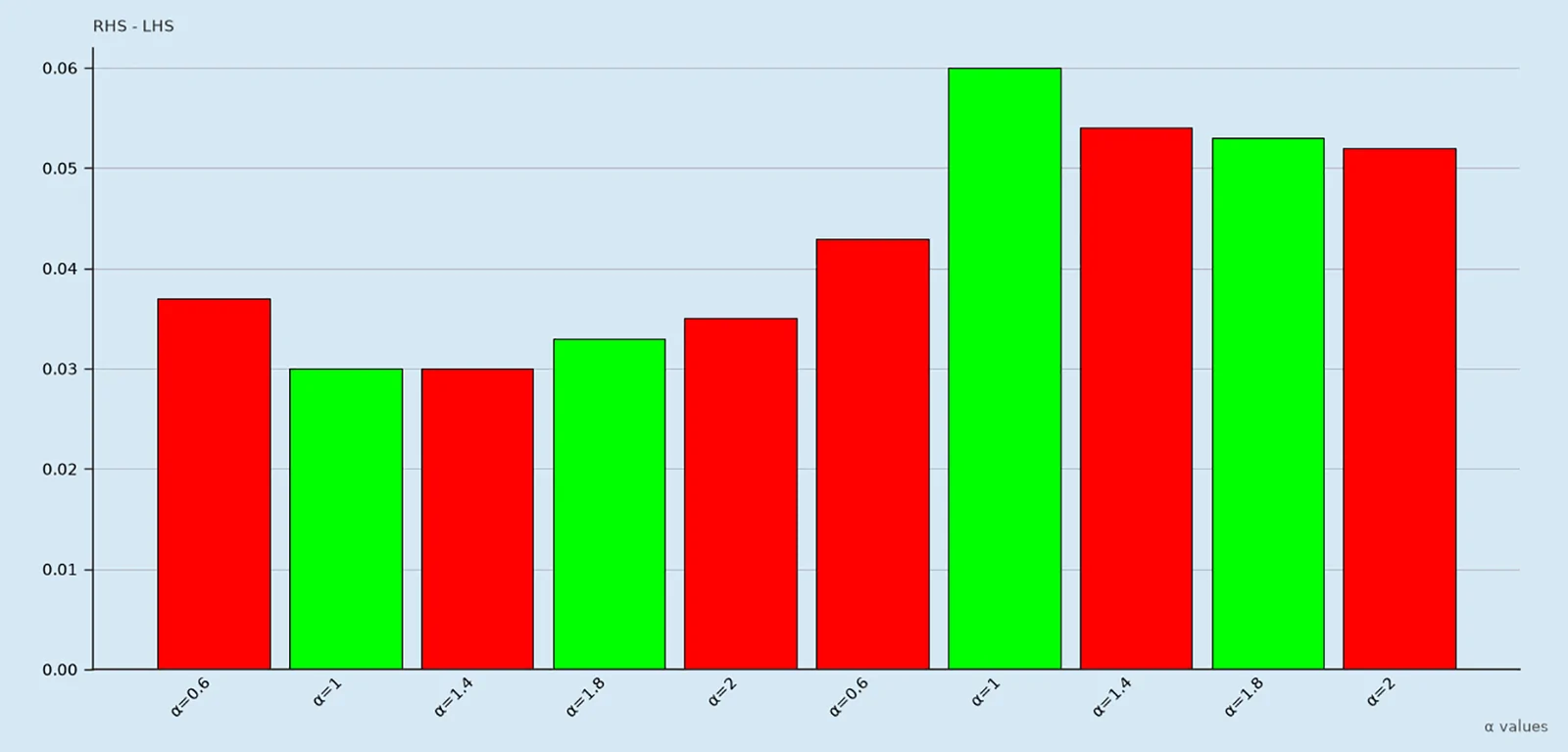
Bar chart for various α values. Red bars: difference from our inequality (9); green bars: difference from inequality (12) of [25]. All bars are positive (inequality holds). Red bars are shorter, confirming better performance. Horizontal axis: α, vertical axis: RHS-LHS.

The contour plots in Fig 9 confirm that our inequality (9) provides tighter bounds than inequality (12) of [25]. The red contours (our inequality) lie below the green contours (inequality (12) of [25]) across most of the parameter space, demonstrating the superior performance of our results.

(a) Our Theorem 3.10
(b) Theorem 5 of [25]
(c) Difference

**Fig 9:**
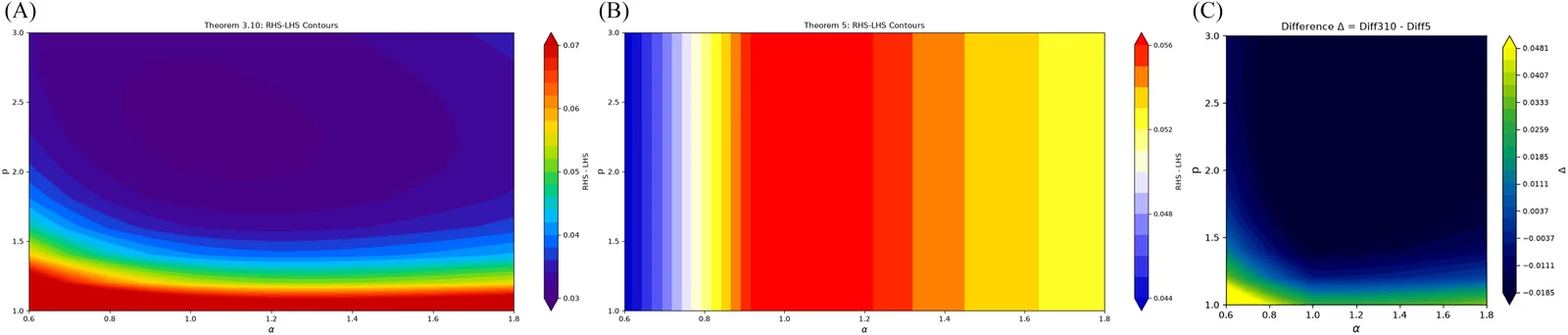
Comparative contour plots: (a) RHS-LHS for our inequality (9), (b) RHS-LHS for inequality (12) of [25], (c) difference between the two bounds. Axes: α (horizontal), *p* (vertical). Our inequality yields tighter bounds in most of the parameter region.

## 4 Fractional newton-type inequalities for functions of bounded variation

In this section, we extend our study to functions of bounded variation. Using the Riemann-Liouville fractional integrals, we prove a fractional Newton-type inequality that generalizes some classical results. The proof relies on a piecewise-defined mapping and properties of the Riemann-Stieltjes integral.

**Theorem 4.1.** *Let* $ϝ:[\kappa_{1},\kappa_{2}]\subseteq [0,\infty )\to \mathbb{R}$ *be a function of bounded variation on* $\left[\kappa_{1},\kappa_{2}\right]$*. Then the following fractional Newton-type inequality is obtained.*


$$\begin{array}{cc} & |(3{)}^{p(\alpha -1)}\;\Gamma (\alpha +1)[A_{1}^{\alpha +1}\;{ℑ}_{\kappa_{1}+}^{\alpha }\;ϝ\left(m\left(\frac{2\kappa_{1}+\kappa_{2}}{3}\right)\right)+A_{2}^{\alpha +1}\;{ℑ}_{\frac{2\kappa_{1}+\kappa_{2}}{3}+}^{\alpha }\;ϝ\left(m\left(\frac{\kappa_{1}+2\kappa_{2}}{3}\right)\right)\\ & +A_{3}^{\alpha +1}\;{ℑ}_{\frac{\kappa_{1}+2\kappa_{2}}{3}+}^{\alpha }\;ϝ\left(m\kappa_{2}\right)]-\frac{1}{8}[3A_{1}ϝ(\kappa_{1})+5A_{1}\times ϝ\left(m\left(\frac{2\kappa_{1}+\kappa_{2}}{3}\right)\right)+4A_{2}\times \\ & ϝ\left(m\left(\frac{2\kappa_{1}+\kappa_{2}}{3}\right)\right)+4A_{2}\times ϝ\left(m\left(\frac{\kappa_{1}+2\kappa_{2}}{3}\right)\right)+5A_{3}\times ϝ\left(\left(\frac{\kappa_{1}+2\kappa_{2}}{3}\right)\right)+3A_{3}\times ϝ(m\kappa_{2})]|\\ & \leq \frac{5}{8}\left[A_{1}\overset{\frac{2\kappa_{1}+\kappa_{2}}{3}}{\underset{\kappa_{1}}{⋁}}(ϝ)+A_{2}\overset{\frac{\kappa_{1}+2\kappa_{2}}{3}}{\underset{\frac{2\kappa_{1}+\kappa_{2}}{3}}{⋁}}(ϝ)+A_{3}\overset{\kappa_{2}}{\underset{\frac{\kappa_{1}+2\kappa_{2}}{3}}{⋁}}(ϝ)\right], \end{array}$$ (11)

*where A*_*1*_*, A*_*2*_*, and A*_*3*_ *are given in (2) and* $\overset{\kappa_{2}}{\underset{\kappa_{1}}{⋁}}(ϝ)$ *denotes the total variation of* F *on* $\left[\kappa_{1},\kappa_{2}\right]$.

*Proof.* Define a mapping $M_{\alpha }$ as follows


$$\begin{array}{c} M_{\alpha }(\vartheta )=\left\{ \begin{array}{cc} \frac{{\left(m{\left(\frac{2\kappa_{1}+\kappa_{2}}{3}\right)}^{p}-\vartheta^{p}\right)}^{\alpha }-\frac{5}{8}{\left(m{\left(\frac{2\kappa_{1}+\kappa_{2}}{3}\right)}^{p}-\kappa_{1}^{p}\right)}^{\alpha }}{{\left(m{\left(\frac{2\kappa_{1}+\kappa_{2}}{3}\right)}^{p}-\kappa_{1}^{p}\right)}^{\alpha +1}}, & if\;\;\;\kappa_{1}\leq \vartheta \leq \frac{2\kappa_{1}+\kappa_{2}}{3};\}\\ \frac{{\left(m{\left(\frac{\kappa_{1}+2\kappa_{2}}{3}\right)}^{p}-\vartheta^{p}\right)}^{\alpha }-\frac{1}{2}{\left(m{\left(\frac{\kappa_{1}+2\kappa_{2}}{3}\right)}^{p}-{\left(\frac{2\kappa_{1}+\kappa_{2}}{3}\right)}^{p}\right)}^{\alpha }}{{\left(m{\left(\frac{\kappa_{1}+2\kappa_{2}}{3}\right)}^{p}-{\left(\frac{2\kappa_{1}+\kappa_{2}}{3}\right)}^{p}\right)}^{\alpha +1}}, & if\;\;\;\frac{2\kappa_{1}+\kappa_{2}}{3}<\vartheta \leq \frac{\kappa_{1}+2\kappa_{2}}{3};\}\\ \frac{{\left(m\kappa_{2}^{p}-\vartheta^{p}\right)}^{\alpha }-\frac{3}{8}{\left(m\kappa_{2}^{p}-{\left(\frac{\kappa_{1}+2\kappa_{2}}{3}\right)}^{p}\right)}^{\alpha }}{{\left(m\kappa_{2}^{p}-{\left(\frac{\kappa_{1}+2\kappa_{2}}{3}\right)}^{p}\right)}^{\alpha +1}}, & if\;\;\;\frac{\kappa_{1}+2\kappa_{2}}{3}<\vartheta \leq \kappa_{2}.\} \end{array}\right. \end{array}$$

It follows from that


$$\begin{array}{cc} \int_{\kappa_{1}}^{\kappa_{2}}M_{\alpha }(\vartheta )dϝ(\vartheta ) & =\int_{\kappa_{1}}^{\frac{2\kappa_{1}+\kappa_{2}}{3}}\frac{{\left(m{\left(\frac{2\kappa_{1}+\kappa_{2}}{3}\right)}^{p}-\vartheta^{p}\right)}^{\alpha }-\frac{5}{8}{\left(m{\left(\frac{2\kappa_{1}+\kappa_{2}}{3}\right)}^{p}-\kappa_{1}^{p}\right)}^{\alpha }}{{\left(m{\left(\frac{2\kappa_{1}+\kappa_{2}}{3}\right)}^{p}-\kappa_{1}^{p}\right)}^{\alpha +1}}dϝ(\vartheta )\\ & +\int_{\frac{2\kappa_{1}+\kappa_{2}}{3}}^{\frac{\kappa_{1}+2\kappa_{2}}{3}}\frac{{\left(m{\left(\frac{\kappa_{1}+2\kappa_{2}}{3}\right)}^{p}-\vartheta^{p}\right)}^{\frac{\alpha }{k}}-\frac{1}{2}{\left(m{\left(\frac{\kappa_{1}+2\kappa_{2}}{3}\right)}^{p}-{\left(\frac{2\kappa_{1}+\kappa_{2}}{3}\right)}^{p}\right)}^{\alpha }}{{\left(m{\left(\frac{\kappa_{1}+2\kappa_{2}}{3}\right)}^{p}-{\left(\frac{2\kappa_{1}+\kappa_{2}}{3}\right)}^{p}\right)}^{\alpha +1}}dϝ(\vartheta )\\ & +\int_{\frac{\kappa_{1}+2\kappa_{2}}{3}}^{\kappa_{2}}\frac{{\left(m\kappa_{2}^{p}-\vartheta^{p}\right)}^{\alpha }-\frac{3}{8}{\left(m\kappa_{2}^{p}-{\left(\frac{\kappa_{1}+2\kappa_{2}}{3}\right)}^{p}\right)}^{\alpha }}{{\left(m\kappa_{2}^{p}-{\left(\frac{\kappa_{1}+2\kappa_{2}}{3}\right)}^{p}\right)}^{\alpha +1}}dϝ(\vartheta )\\ & =I_{1}+I_{2}+I_{3}. \end{array}$$ (12)

Integrating by parts


$$\begin{array}{cc} & I_{1}=\int_{\kappa_{1}}^{\frac{2\kappa_{1}+\kappa_{2}}{3}}\frac{{\left(m{\left(\frac{2\kappa_{1}+\kappa_{2}}{3}\right)}^{p}-\vartheta^{p}\right)}^{\alpha }-\frac{5}{8}{\left(m{\left(\frac{2\kappa_{1}+\kappa_{2}}{3}\right)}^{p}-\kappa_{1}^{p}\right)}^{\alpha }}{{\left(m{\left(\frac{2\kappa_{1}+\kappa_{2}}{3}\right)}^{p}-\kappa_{1}^{p}\right)}^{\alpha +1}}dϝ(\vartheta )\\ & =-\frac{5p}{8\left(m{\left(\frac{2\kappa_{1}+\kappa_{2}}{3}\right)}^{p}-\kappa_{1}^{p}\right)}ϝ\left(m\left(\frac{2\kappa_{1}+\kappa_{2}}{3}\right)\right)-\frac{3p}{8\left(m{\left(\frac{2\kappa_{1}+\kappa_{2}}{3}\right)}^{p}-\kappa_{1}^{p}\right)}ϝ(\kappa_{1})\\ & +\frac{1}{{\left(m{\left(\frac{2\kappa_{1}+\kappa_{2}}{3}\right)}^{p}-\kappa_{1}^{p}\right)}^{\alpha +1}}(p{)}^{2}\Gamma (\alpha +1)\;{ℑ}_{\kappa_{1}+}^{\alpha }\;ϝ\left(m\left(\frac{2\kappa_{1}+\kappa_{2}}{3}\right)\right), \end{array}$$ (13)

Similarly


$$\begin{array}{cc} & I_{2}=\int_{\frac{2\kappa_{1}+\kappa_{2}}{3}}^{\frac{\kappa_{1}+2\kappa_{2}}{3}}\frac{{\left(m{\left(\frac{\kappa_{1}+2\kappa_{2}}{3}\right)}^{p}-\vartheta^{p}\right)}^{\alpha }-\frac{1}{2}{\left(m{\left(\frac{\kappa_{1}+2\kappa_{2}}{3}\right)}^{p}-{\left(\frac{2\kappa_{1}+\kappa_{2}}{3}\right)}^{p}\right)}^{\alpha }}{{\left(m{\left(\frac{\kappa_{1}+2\kappa_{2}}{3}\right)}^{p}-{\left(\frac{2\kappa_{1}+\kappa_{2}}{3}\right)}^{p}\right)}^{\alpha +1}}dϝ(\vartheta )\\ & =-\frac{p}{2\left(m{\left(\frac{\kappa_{1}+2\kappa_{2}}{3}\right)}^{p}-{\left(\frac{2\kappa_{1}+\kappa_{2}}{3}\right)}^{p}\right)}ϝ\left(m\left(\frac{\kappa_{1}+2\kappa_{2}}{3}\right)\right)-\frac{p}{2\left(m{\left(\frac{\kappa_{1}+2\kappa_{2}}{3}\right)}^{p}-{\left(\frac{2\kappa_{1}+\kappa_{2}}{3}\right)}^{p}\right)}\\ & \times ϝ\left(\frac{2\kappa_{1}+\kappa_{2}}{3}\right)+\frac{1}{{\left(m{\left(\frac{\kappa_{1}+2\kappa_{2}}{3}\right)}^{p}-{\left(\frac{2\kappa_{1}+\kappa_{2}}{3}\right)}^{p}\right)}^{\alpha +1}}(p{)}^{2}\Gamma (\alpha +1)\\ & \times {ℑ}_{\frac{2\kappa_{1}+\kappa_{2}}{3}+}^{\alpha }\;ϝ\left(m\left(\frac{\kappa_{1}+2\kappa_{2}}{3}\right)\right), \end{array}$$ (14)

and


$$\begin{array}{cc} & I_{3}=\int_{\frac{\kappa_{1}+2\kappa_{2}}{3}}^{\kappa_{2}}\frac{{\left(m\kappa_{2}^{p}-\vartheta^{p}\right)}^{\alpha }-\frac{3}{8}{\left(m\kappa_{2}^{p}-{\left(\frac{\kappa_{1}+2\kappa_{2}}{3}\right)}^{p}\right)}^{\alpha }}{{\left(m\kappa_{2}^{p}-{\left(\frac{\kappa_{1}+2\kappa_{2}}{3}\right)}^{p}\right)}^{\alpha +1}}dϝ(\vartheta )\\ & =-\frac{3}{8\left(m\kappa_{2}^{p}-{\left(\frac{\kappa_{1}+2\kappa_{2}}{3}\right)}^{p}\right)}ϝ\left(m\kappa_{2}\right)-\frac{5}{8\left(m\kappa_{2}^{p}-{\left(\frac{\kappa_{1}+2\kappa_{2}}{3}\right)}^{p}\right)}ϝ\left(\frac{\kappa_{1}+2\kappa_{2}}{3}\right)\\ & +\frac{1}{{\left(m\kappa_{2}^{p}-{\left(\frac{\kappa_{1}+2\kappa_{2}}{3}\right)}^{p}\right)}^{\alpha +1}}(p{)}^{2}\Gamma (\alpha +1)\;{ℑ}_{\frac{\kappa_{1}+2\kappa_{2}}{3}+}^{\alpha }\;ϝ\left(m\kappa_{2}\right). \end{array}$$ (15)

Substituting (13)-(15) into (12) yields


$$\begin{array}{cc} & (3{)}^{p(\alpha -1)}\;\Gamma (\alpha +1)[A_{1}^{\alpha +1}\;{ℑ}_{\kappa_{1}+}^{\alpha }\;ϝ\left(m\left(\frac{2\kappa_{1}+\kappa_{2}}{3}\right)\right)+A_{2}^{\alpha +1}\;{ℑ}_{\frac{2\kappa_{1}+\kappa_{2}}{3}+}^{\alpha }\;ϝ\left(m\left(\frac{\kappa_{1}+2\kappa_{2}}{3}\right)\right)\\ & +A_{3}^{\alpha +1}\;{ℑ}_{\frac{\kappa_{1}+2\kappa_{2}}{3}+}^{\alpha }\;ϝ\left(m\kappa_{2}\right)]-\frac{1}{8}[3A_{1}ϝ(\kappa_{1})+5A_{1}\times ϝ\left(m\left(\frac{2\kappa_{1}+\kappa_{2}}{3}\right)\right)+4A_{2}\times ϝ\left(\frac{2\kappa_{1}+\kappa_{2}}{3}\right)\\ & +4A_{2}\times ϝ\left(m\left(\frac{\kappa_{1}+2\kappa_{2}}{3}\right)\right)+5A_{3}\times ϝ\left(\frac{\kappa_{1}+2\kappa_{2}}{3}\right)+3A_{3}\times ϝ(m\kappa_{2})]\\ & =\frac{1}{3^{p}}\int_{\kappa_{1}}^{\kappa_{2}}M_{\alpha }(\vartheta )dϝ(\vartheta ). \end{array}$$

It is well known that if $\Psi ,ϝ:[\kappa_{1},\kappa_{2}]\to \mathbb{R}$ are such that Ψ is continuous on $\left[\kappa_{1},\kappa_{2}\right]$ and ϝ is of bounded variation on $\left[\kappa_{1},\kappa_{2}\right]$, then $\int_{\kappa_{1}}^{\kappa_{2}}\Psi (\delta )dϝ(\delta )$ exists and


$$\begin{array}{c} \left|\int_{\kappa_{1}}^{\kappa_{2}}\Psi (\delta )dϝ(\delta )\right|\leq \sup_{\delta \in [\kappa_{1},\kappa_{2}]}|\Psi (\delta )|\overset{\kappa_{2}}{\underset{\kappa_{1}}{⋁}}(ϝ). \end{array}$$ (16)

On the other hand, using (16), we obtain


$$\begin{array}{cc} & |(3{)}^{p(\alpha -1)}\;\Gamma (\alpha +1)[A_{1}^{\alpha +1}\;{ℑ}_{\kappa_{1}+}^{\alpha }\;ϝ\left(m\left(\frac{2\kappa_{1}+\kappa_{2}}{3}\right)\right)+A_{2}^{\alpha +1}\;{ℑ}_{\frac{2\kappa_{1}+\kappa_{2}}{3}+}^{\alpha }\;ϝ\left(m\left(\frac{\kappa_{1}+2\kappa_{2}}{3}\right)\right)\\ & +A_{3}^{\alpha +1}\;{ℑ}_{\frac{\kappa_{1}+2\kappa_{2}}{3}+}^{\alpha }\;ϝ\left(m\kappa_{2}\right)]-\frac{1}{8}[3A_{1}ϝ(\kappa_{1})+5A_{1}\times ϝ\left(m\left(\frac{2\kappa_{1}+\kappa_{2}}{3}\right)\right)+4A_{2}\times ϝ\left(\frac{2\kappa_{1}+\kappa_{2}}{3}\right)\\ & +4A_{2}\times ϝ\left(m\left(\frac{\kappa_{1}+2\kappa_{2}}{3}\right)\right)+5A_{3}\times ϝ\left(\frac{\kappa_{1}+2\kappa_{2}}{3}\right)+3A_{3}\times ϝ(m\kappa_{2})]|\\ & =\frac{1}{3^{p}}\left|\int_{\kappa_{1}}^{\kappa_{2}}M_{\alpha }(\vartheta )dϝ(\vartheta )\right| \end{array}$$

$$\begin{array}{cc} & \leq \frac{1}{3^{p}}[\left|\int_{\kappa_{1}}^{\frac{2\kappa_{1}+\kappa_{2}}{3}}\frac{{\left(m{\left(\frac{2\kappa_{1}+\kappa_{2}}{3}\right)}^{p}-\vartheta^{p}\right)}^{\alpha }-\frac{5}{8}{\left(m{\left(\frac{2\kappa_{1}+\kappa_{2}}{3}\right)}^{p}-\kappa_{1}^{p}\right)}^{\alpha }}{{\left(m{\left(\frac{2\kappa_{1}+\kappa_{2}}{3}\right)}^{p}-\kappa_{1}^{p}\right)}^{\alpha +1}}dϝ(\vartheta )\right|\\ & +\left|\int_{\frac{2\kappa_{1}+\kappa_{2}}{3}}^{\frac{\kappa_{1}+2\kappa_{2}}{3}}\frac{{\left(m{\left(\frac{\kappa_{1}+2\kappa_{2}}{3}\right)}^{p}-\vartheta^{p}\right)}^{\alpha }-\frac{1}{2}{\left(m{\left(\frac{\kappa_{1}+2\kappa_{2}}{3}\right)}^{p}-{\left(\frac{2\kappa_{1}+\kappa_{2}}{3}\right)}^{p}\right)}^{\alpha }}{{\left(m{\left(\frac{\kappa_{1}+2\kappa_{2}}{3}\right)}^{p}-{\left(\frac{2\kappa_{1}+\kappa_{2}}{3}\right)}^{p}\right)}^{\alpha +1}}dϝ(\vartheta )\right|\\ & +\left|\int_{\frac{\kappa_{1}+2\kappa_{2}}{3}}^{\kappa_{2}}\frac{{\left(m\kappa_{2}^{p}-\vartheta^{p}\right)}^{\alpha }-\frac{3}{8}{\left(m\kappa_{2}^{p}-{\left(\frac{\kappa_{1}+2\kappa_{2}}{3}\right)}^{p}\right)}^{\alpha }}{{\left(m\kappa_{2}^{p}-{\left(\frac{\kappa_{1}+2\kappa_{2}}{3}\right)}^{p}\right)}^{\alpha +1}}dϝ(\vartheta )\right|] \end{array}$$

$$\begin{array}{cc} & \leq \frac{1}{3^{p}}[\sup_{\vartheta \in \left[\kappa_{1},\frac{2\kappa_{1}+\kappa_{2}}{3}\right]}\left|\frac{{\left(m{\left(\frac{2\kappa_{1}+\kappa_{2}}{3}\right)}^{p}-\vartheta^{p}\right)}^{\alpha }-\frac{5}{8}{\left(m{\left(\frac{2\kappa_{1}+\kappa_{2}}{3}\right)}^{p}-\kappa_{1}^{p}\right)}^{\alpha }}{{\left(m{\left(\frac{2\kappa_{1}+\kappa_{2}}{3}\right)}^{p}-\kappa_{1}^{p}\right)}^{\alpha +1}}\right|\overset{\frac{2\kappa_{1}+\kappa_{2}}{3}}{\underset{\kappa_{1}}{⋁}}(ϝ)\\ & +\sup_{\vartheta \in \left[\frac{2\kappa_{1}+\kappa_{2}}{3},\frac{\kappa_{1}+2\kappa_{2}}{3}\right]}\left|\frac{{\left(m{\left(\frac{\kappa_{1}+2\kappa_{2}}{3}\right)}^{p}-\vartheta^{p}\right)}^{\alpha }-\frac{1}{2}{\left(m{\left(\frac{\kappa_{1}+2\kappa_{2}}{3}\right)}^{p}-{\left(\frac{2\kappa_{1}+\kappa_{2}}{3}\right)}^{p}\right)}^{\alpha }}{{\left(m{\left(\frac{\kappa_{1}+2\kappa_{2}}{3}\right)}^{p}-{\left(\frac{2\kappa_{1}+\kappa_{2}}{3}\right)}^{p}\right)}^{\alpha +1}}\right|\overset{\frac{\kappa_{1}+2\kappa_{2}}{3}}{\underset{\frac{2\kappa_{1}+\kappa_{2}}{3}}{⋁}}(ϝ)\\ & +\sup_{\vartheta \in \left[\frac{\kappa_{1}+2\kappa_{2}}{3},\kappa_{2}\right]}\left|\frac{{\left(m\kappa_{2}^{p}-\vartheta^{p}\right)}^{\alpha }-\frac{3}{8}{\left(m\kappa_{2}^{p}-{\left(\frac{\kappa_{1}+2\kappa_{2}}{3}\right)}^{p}\right)}^{\alpha }}{{\left(m\kappa_{2}^{p}-{\left(\frac{\kappa_{1}+2\kappa_{2}}{3}\right)}^{p}\right)}^{\alpha +1}}\right|\overset{\kappa_{2}}{\underset{\frac{\kappa_{1}+2\kappa_{2}}{3}}{⋁}}(ϝ)] \end{array}$$

$$\begin{array}{cc} & =\frac{1}{3^{p}}[\frac{5}{8\left(m{\left(\frac{2\kappa_{1}+\kappa_{2}}{3}\right)}^{p}-\kappa_{1}^{p}\right)}\overset{\frac{2\kappa_{1}+\kappa_{2}}{3}}{\underset{\kappa_{1}}{⋁}}(ϝ)+\frac{1}{2\left(m{\left(\frac{\kappa_{1}+2\kappa_{2}}{3}\right)}^{p}-{\left(\frac{2\kappa_{1}+\kappa_{2}}{3}\right)}^{p}\right)}\overset{\frac{\kappa_{1}+2\kappa_{2}}{3}}{\underset{\frac{2\kappa_{1}+\kappa_{2}}{3}}{⋁}}(ϝ)\\ & +\frac{3}{8\left(m\kappa_{2}^{p}-{\left(\frac{\kappa_{1}+2\kappa_{2}}{3}\right)}^{p}\right)}\overset{\frac{\kappa_{1}+2\kappa_{2}}{3}}{\underset{\frac{2\kappa_{1}+\kappa_{2}}{3}}{⋁}}(ϝ)]\\ & \leq \frac{5}{8}\left[A_{1}\overset{\frac{2\kappa_{1}+\kappa_{2}}{3}}{\underset{\kappa_{1}}{⋁}}(ϝ)+A_{2}\overset{\frac{\kappa_{1}+2\kappa_{2}}{3}}{\underset{\frac{2\kappa_{1}+\kappa_{2}}{3}}{⋁}}(ϝ)+A_{3}\overset{\frac{\kappa_{1}+2\kappa_{2}}{3}}{\underset{\frac{2\kappa_{1}+\kappa_{2}}{3}}{⋁}}(ϝ)\right], \end{array}$$

which is the required proof. □

**Remark 5.** *The following relations indicate that Theorem 4.1 presents more general results than the existing literature.*

(i) *By taking p = 1,* α=1*, s = 1, m = 1, and* sinh(p𝔱)=p𝔱*, the corollary of Theorem 2.6 is captured for the choice* $\lambda =\frac{1}{4}$ *and* $x=\frac{2\kappa_{1}+\kappa_{2}}{3}$.
(ii) *By setting p = 1, s = 1, m = 1, and* sinh(p𝔱)=p𝔱*, the corollary of Theorem 2.7 is obtained for the choice* $\phi (\vartheta )=\frac{\vartheta^{\alpha }}{\Gamma (\alpha )}$.
(iii) *By taking p = 1, s = 1, m = 1, and* sinh(p𝔱)=p𝔱*, we have Theorem 7 of [25].*

## 5 Conclusions

This paper explores new variants of Newton-type inequalities for differentiable modified hyperbolic *p*-convex functions and functions of bounded variation, using Riemann-Liouville fractional integrals. Some refined results are obtained by applying power-mean and Hölder inequalities. Numerical examples and 2D and 3D graphical representations support the findings, and a comparative analysis with existing work illustrates that our findings generalize and enhance already existing inequalities. Future research areas may include the application of these findings to additional fractional derivatives such as Caputo and Atangana-Baleanu derivatives, proving error estimates for numerical methods of fractional integrals, the study of similar inequalities in quantum calculus, and further applications in machine learning.
